# Supplementary material for: A gut microbiome-kidney-heart axis predictive of future cardiovascular diseases
Source: Nat Commun. 2026 Mar 5;17:3477. doi: 10.1038/s41467-026-69405-0 (PMC13079829; doi:10.1038/s41467-026-69405-0)
Supplement: Supplementary file 1 — Supplementary Information [file 41467_2026_69405_MOESM1_ESM.pdf]

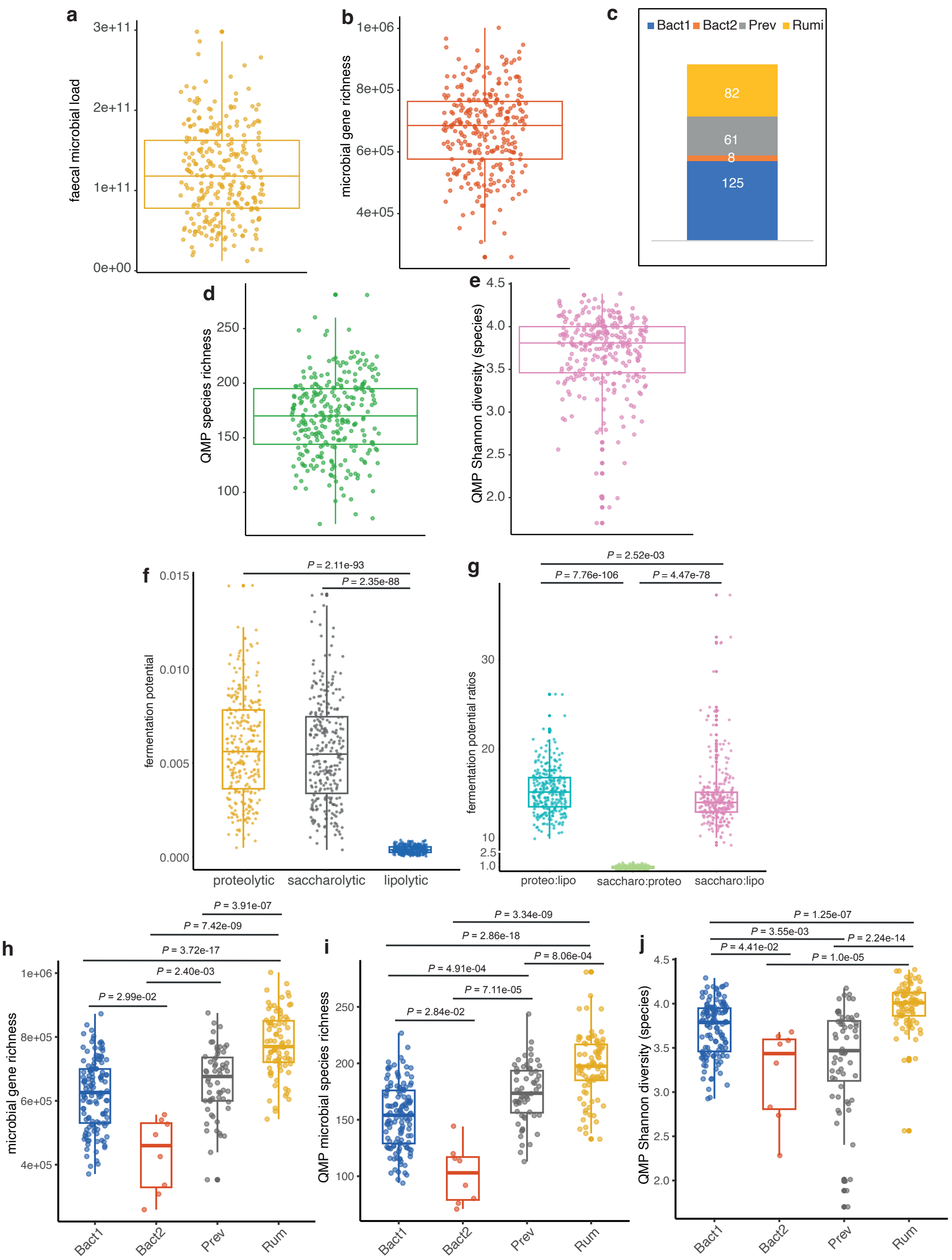

Supplementary Fig. 1. Metagenomic features of the metabolically healthy individuals (n = 275). Distribution of **a**, faecal microbial load, **b**, microbial gene richness, **c**, enterotypes, **d,** microbial species richness **e,** Shannon species diversity, **f**, saccharolytic-, proteolytic- and lipolytic- fermentation potential, and **g**, their respective ratios in the MetaCardis healthy individuals. *P*-values were derived from Kruskal-Wallis with Dunn-Bonferroni *post hoc* tests. Next, we tested the distribution of these variables based on enterotypes and only those exhibiting significant differences are shown here. Distribution of **h**, microbial gene richness **i,** Shannon species richness, and **j,** microbial species diversity based on enterotypes in the metabolically healthy individuals (Kruskal-Wallis with Dunn-Bonferroni *post hoc* tests). Bact1, *Bacteroides 1* enterotype; Bact2, *Bacteroides 2* enterotype; Prev, *Prevotella* enterotype; Rumi, *Ruminococcus* enterotype; QMP, quantitative microbiome profiling.

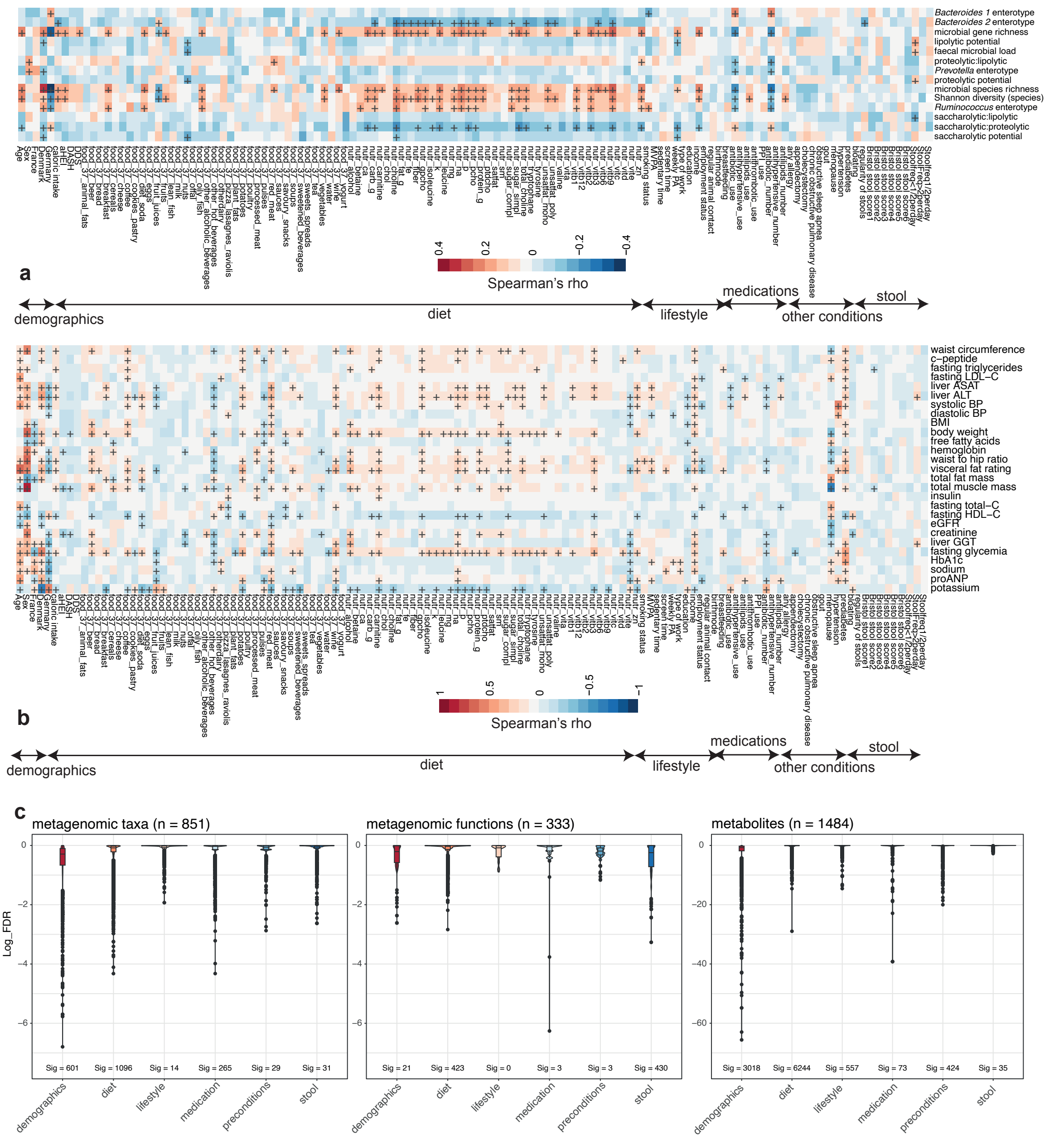

Supplementary Fig. 2. Host variables associate with microbial taxonomic and functional features, metabolome and clinical variables in healthy individuals. Heatmaps representing associations among 117 host variables covering diet, drugs, preconditions, demographics, lifestyle and stool characteristics (Supplementary Data 2) with **a**, broad metagenomic and ecological descriptors (n = 229 – 275), and **b**, clinical variables in the MetaCardis healthy individuals (n = 212 - 275); two-sided Spearman rank correlations; multiple testing correction using BH criteria, + represents FDR < 0.1). **c**, Violin plots representing distributions of the log-transformed FDR-values derived from two-sided Spearman rank correlations testing the associations among host variables and individual gut microbial and metabolomics features (i.e., taxa including genus (n = 45) and MGS (n =806), functions including GMM (n= 116) and KEGG pathways (n = 217) and 1484 metabolites) in healthy individuals (n= 201-275; multiple testing correction was done using BH-criteria and FDR < 0.1 was considered significant). For exact sample sizes, see Source Data File; Source data are provided as a Source Data file. aHEI, alternative healthy eating index; DASH, Dietary approaches to stopping hypertension; DDS, Dietary diversity score; MVPA, moderate-to-vigorous physical activity; BH, Benjamini-Hochberg; FDR, false-discovery rate; QMP, quantitative microbiome profiling; MGS, metagenomic species; GMM, gut metabolic modules.

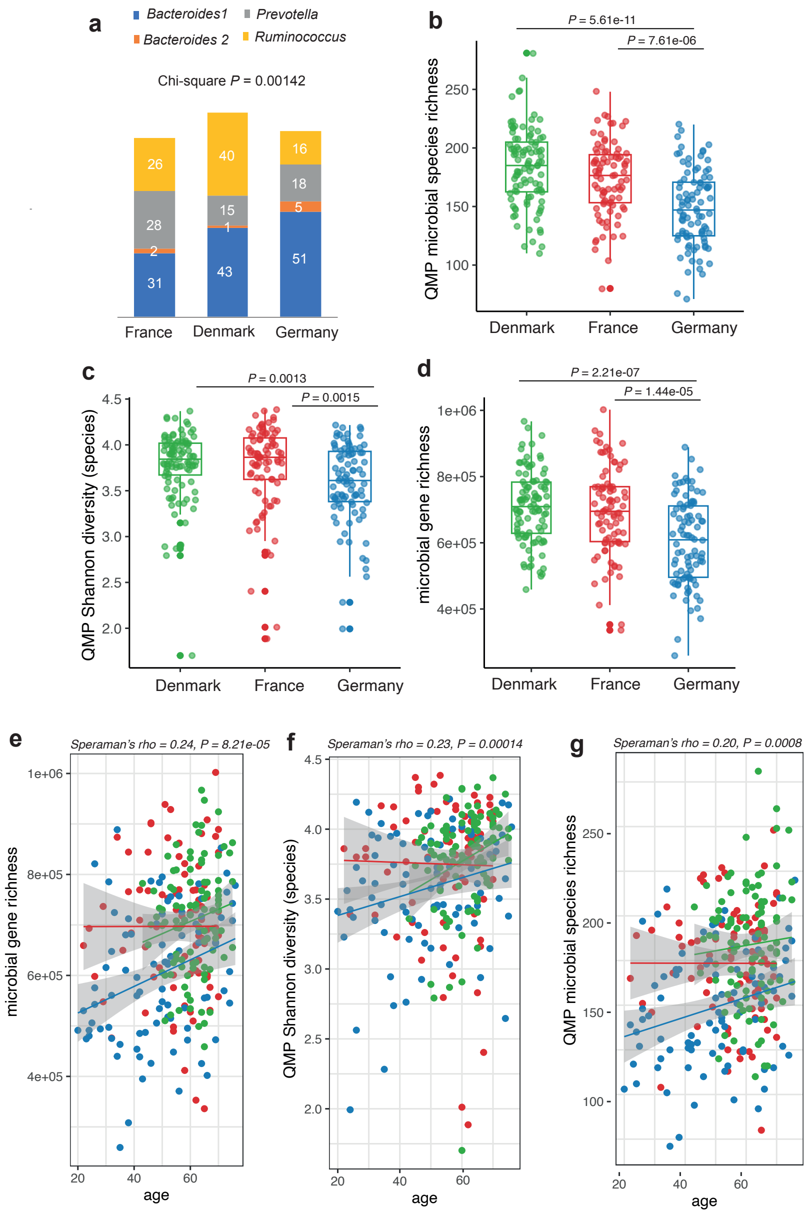

Supplementary Fig. 3. Demographics affects gut microbial features in healthy individuals (n = 275). **a**, Stacked bar plots showing enterotype distribution per country of recruitment *i.e.,* France, Germany and Denmark (Chi-square test). Box plots showing the distribution of **b**, species richness **c**, Shannon species diversity and **d**, gene richness as per country of recruitment (Kruskal-Wallis with Dunn-Bonferroni *post hoc* tests). The body of the boxplot represents the first and third quartiles of the distribution with the median line, and the whiskers extend from the quartiles to the last data point within 1.5× interquartile range with outliers beyond. Scatter plots showing associations between **e**, microbial gene richness **f**, Shannon species diversity and **g**, microbial species richness with age, respectively (two-sided Spearman rank correlations; lines represent distribution per country of recruitment). Only significant associations (FDR <0.1) identified in Supplementary Fig. 2a are plotted here**.** QMP, quantitative microbiome profiling.

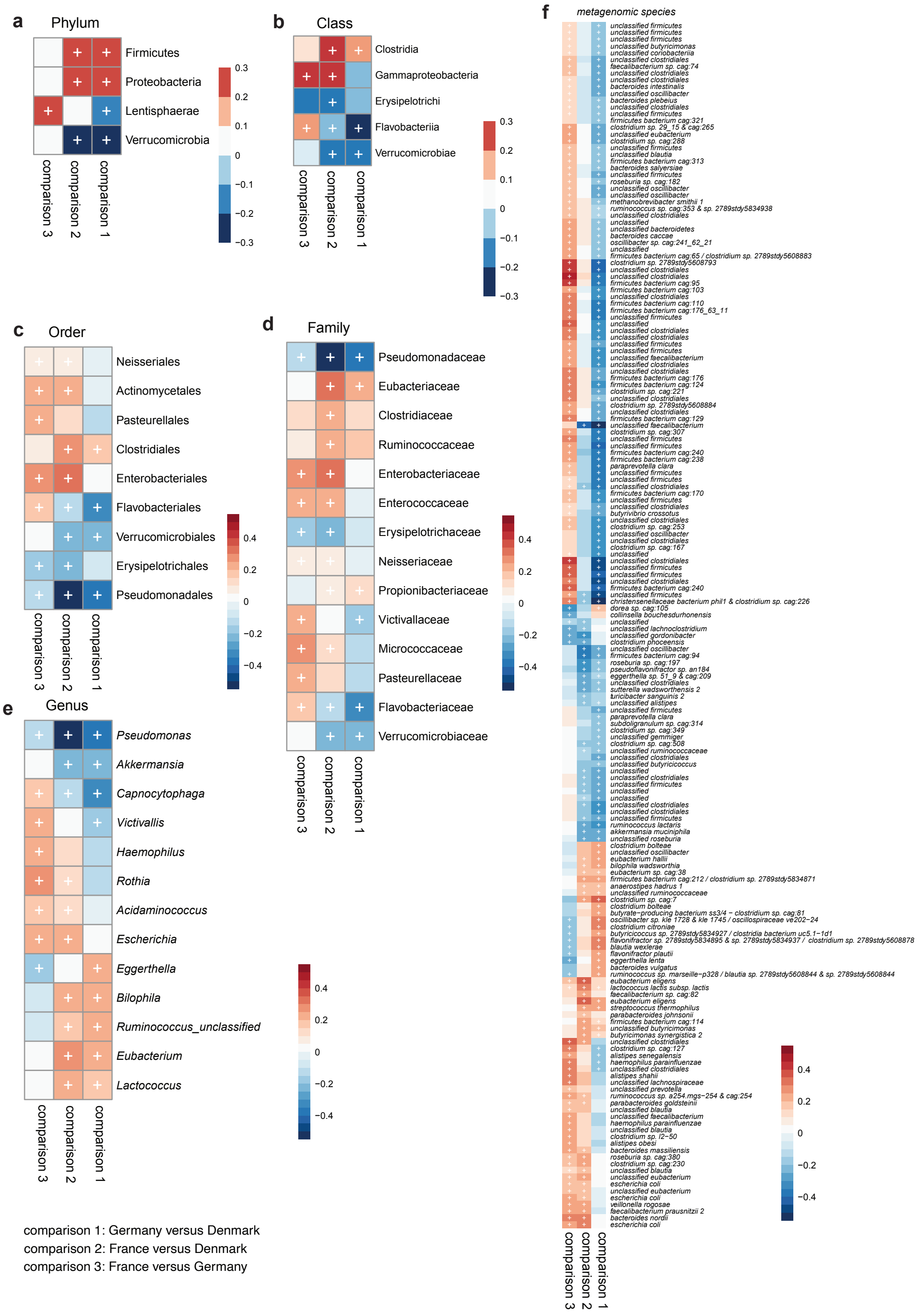

Supplementary Fig. 4. Impact of country of participant recruitment on the gut microbial composition (n = 275). Heatmaps showing the differences in the gut microbiome composition at the level of a) Phylum b) Class, c) Order, d) Family, e) Genus and f) Species among participants recruited from France, Germany and Denmark within the healthy group (n = 275). Effect sizes were calculated using Cliff's Delta. *P*-values were derived from Kruskal-Wallis adjusted for multiple comparisons using the BH criteria. Only variables exhibiting $FDR_{KW} < 0.1$ were subjected to Dunn's pairwise comparisons adjusted for multiple comparisons using the BH criteria. + represents significance as FDR < 0.1 for each pairwise comparison. Source data are provided as a Source Data file.

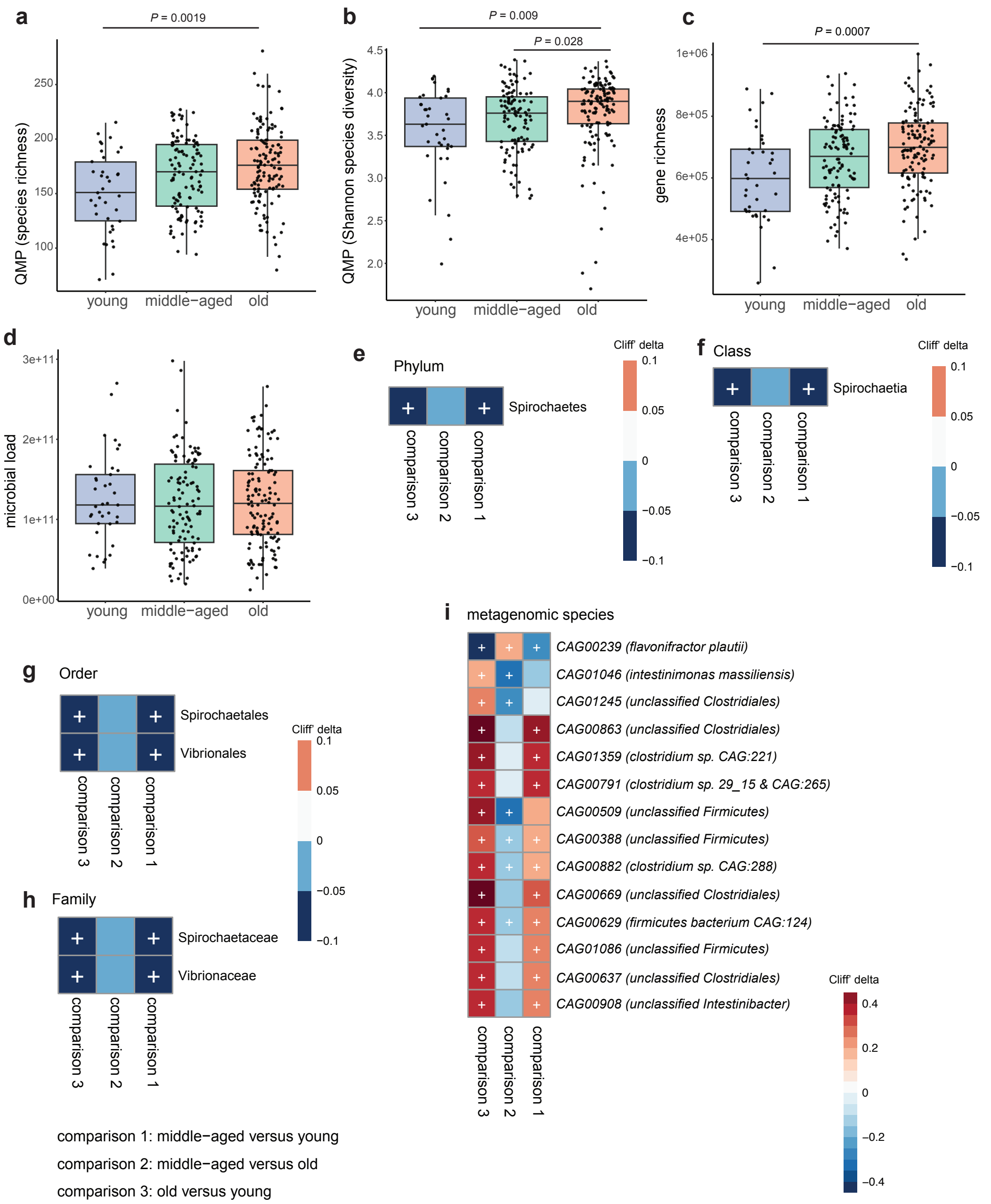

Supplementary Fig. 5. Impact of participant age on the gut microbial composition (n = 275). Box plots showing the differences in the **a,** microbial species richness, **b,** Shannon's species diversity, **c,** microbial gene richness and **d,** fecal microbial load in the healthy group participants when categorized as young (i.e., 20-40 years of age), middle-aged (i.e., 41-59 years of age) and old (i.e., 60-76 years of age). The body of the boxplot represents the first and third quartiles of the distribution with the median line, and the whiskers extend from the quartiles to the last data point within 1.5× interquartile range with outliers beyond. *P*-values were derived from Kruskal-Wallis followed by Dunn's *posthoc* tests. Heatmaps showing the impact of age of participants on the gut microbiome composition at the level of **e,** Phylum, **f,** Class, **g,** Order, **h,** Family and **i,** Species among the healthy individuals (n = 275). Effect sizes were calculated using Cliff's Delta. *P*-values were derived from Kruskal-Wallis adjusted for multiple comparisons using the BH criteria. Only variables exhibiting FDR_{KW} < 0.1 were subjected to Dunn's pairwise comparisons, which were also adjusted for multiple comparisons using the BH criteria (n = 275). + represents significance as FDR < 0.1 for each pairwise comparison. Source data are provided as a Source Data file.

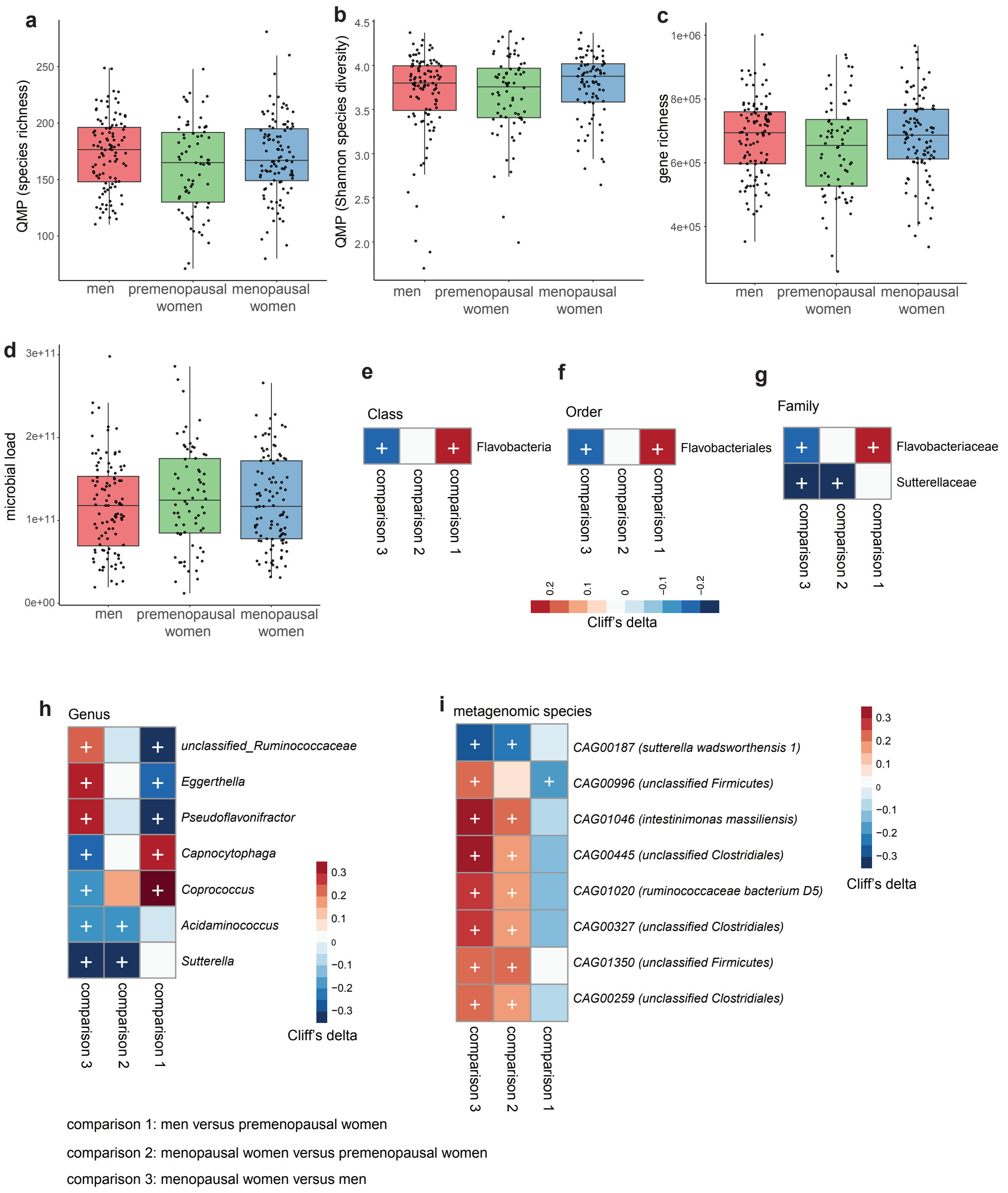

Supplementary Fig. 6. Impact of participant sex and menopausal status on the gut microbial composition (n = 275). Box plots showing the differences in the **a,** microbial species richness, **b,** Shannon's species diversity, **c,** microbial gene richness and **d,** fecal microbial load among men, premenopausal women and post-menopausal women within the healthy group (n = 275).The body of the boxplot represents the first and third quartiles of the distribution with the median line, and the whiskers extend from the quartiles to the last data point within 1.5× interquartile range with outliers beyond. *P*-values were derived from Kruskal-Wallis followed by Dunn's *posthoc* tests. Heatmaps showing the differences in the gut microbiome composition at the level of **e,** Class, **f,** Order, **g,** Family **h,** Genus and **i,** Species among men, premenopausal women and post-menopausal women within the metabolically healthy group (n = 275). Effect sizes were calculated using Cliff's Delta. *P*-values were derived from Kruskal-Wallis adjusted for multiple comparisons using the BH criteria. Only variables exhibiting FDR_{KW} < 0.1 were subjected to Dunn's pairwise comparisons adjusted for multiple comparisons using the BH criteria (n = 275). + represents significance as FDR < 0.1 for each pairwise comparison. Source data are provided as a Source Data file.

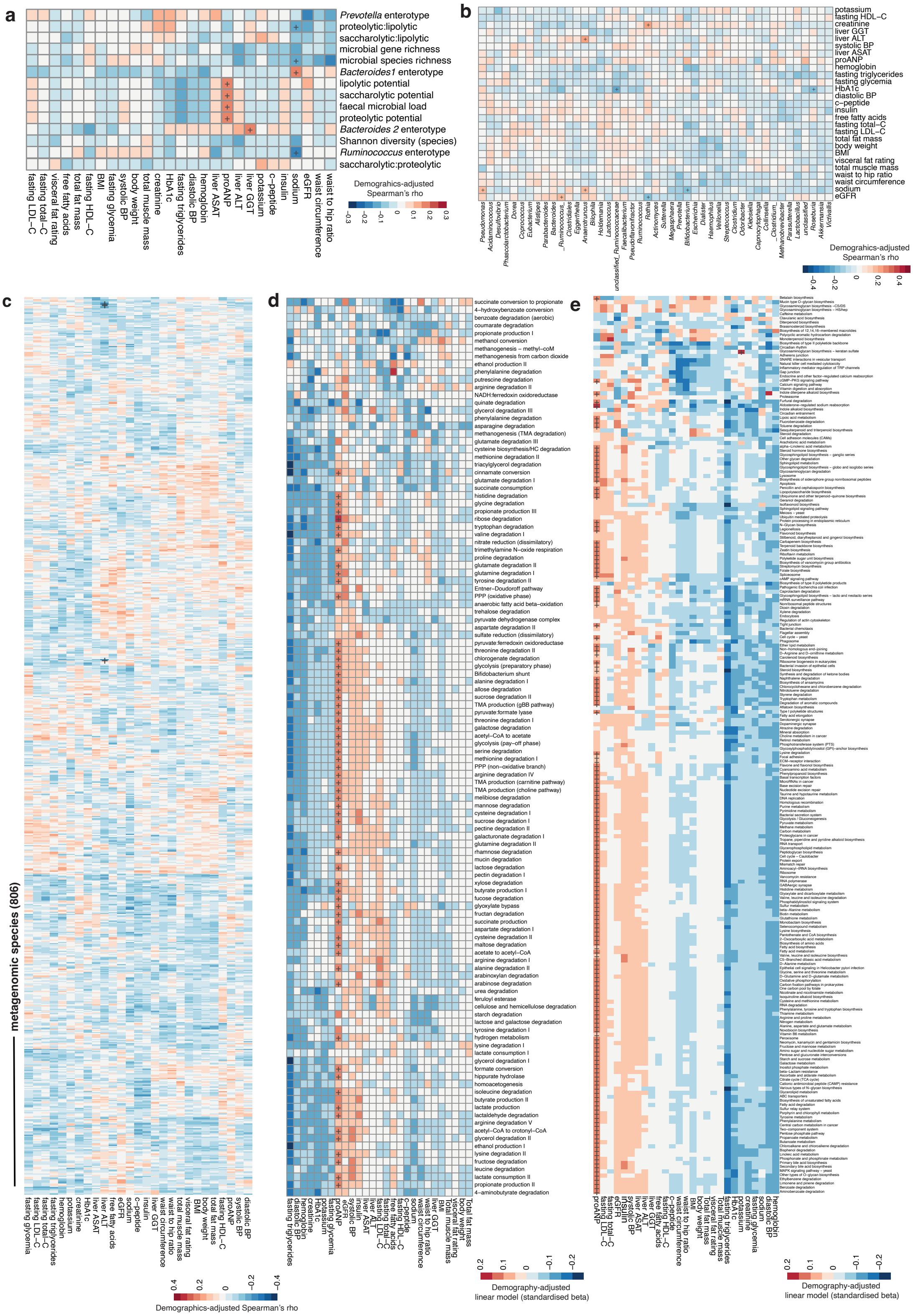

Supplementary Fig. 7. Demographics-adjusted analyses reveal associations among gut microbiome features and circulating proANP in healthy individuals. Heatmaps representing associations among clinical variables with **a**, broad metagenomic descriptors, **b**, genus, **c** MGS, **d**, GMM and **e,** KEGG pathways in healthy individuals (n = 255-275; for exact sample sizes, please see Source Data and Supplementary Data 5). Demographics-adjusted two-sided partial Spearman rank correlation was used for broader metagenome, genus and MGS data whereas demographics-adjusted multiple linear regression using rank normalized

data was used for GMM and KEGG pathways. Multiple testing correction was done using BH criteria; + represents significance as FDR < 0.1. MGS, metagenomic species, GMM, gut metabolic modules; BH, Benjamini-Hochberg; FDR, false-discovery rate. For exact sample sizes, see Source Data File; Source data are provided as a Source Data file.

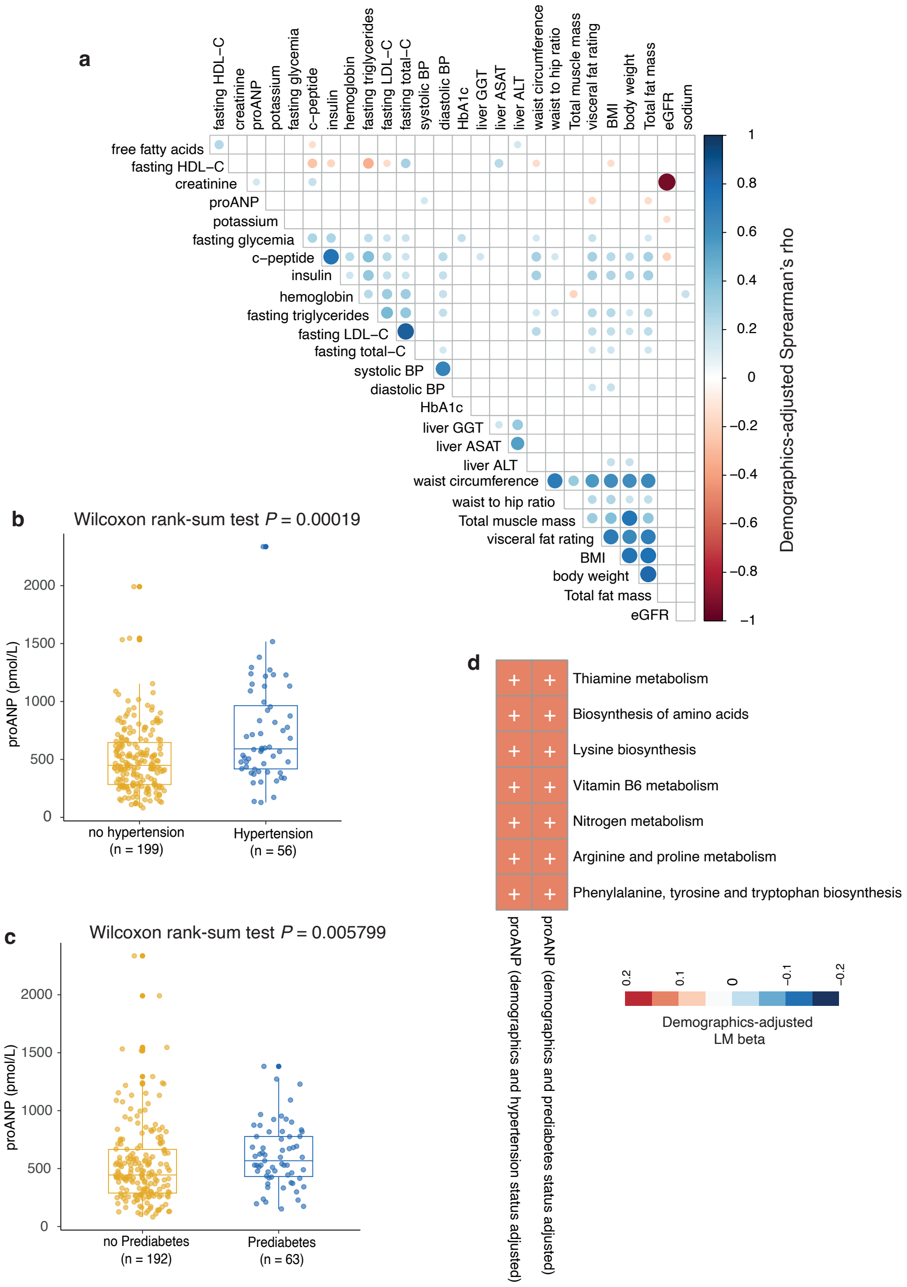

Supplementary Fig. 8. Demographics-adjusted correlates of proANP in healthy individuals. a, Correlogram representing demographics-adjusted two-sided Spearman rank correlations among clinical variables in healthy individuals (n = 247). Only significant associations are shown with size and color of the circle representing effect sizes. Box plots comparing the distribution of circulating proANP levels between **b**, hypertensives versus non-hypertensives, and **c**, pre-diabetics versus non-prediabetics among the MetaCardis healthy individuals (n = 255). The body of the boxplot represents the first and third quartiles of the distribution with the median line, and the whiskers extend from the quartiles to the last data point within 1.5× interquartile range with outliers beyond. *P*-values were derived from the Wilcoxon rank-sum test. **d**, Heatmaps showing seven proANP-associated KEGG pathway features (those partially mediating the relationship between microbial load and circulating proANP levels; FDR_{ACME} < 0.1, Supplementary Data 8) when hypertension and pre-diabetes status were considered as additional covariates to demographics in healthy individuals (n = 255). Multiple linear regression models using rank normalized data; multiple testing correction using BH criteria; represents significance as FDR < 0.1. Source data are provided as a Source Data file.

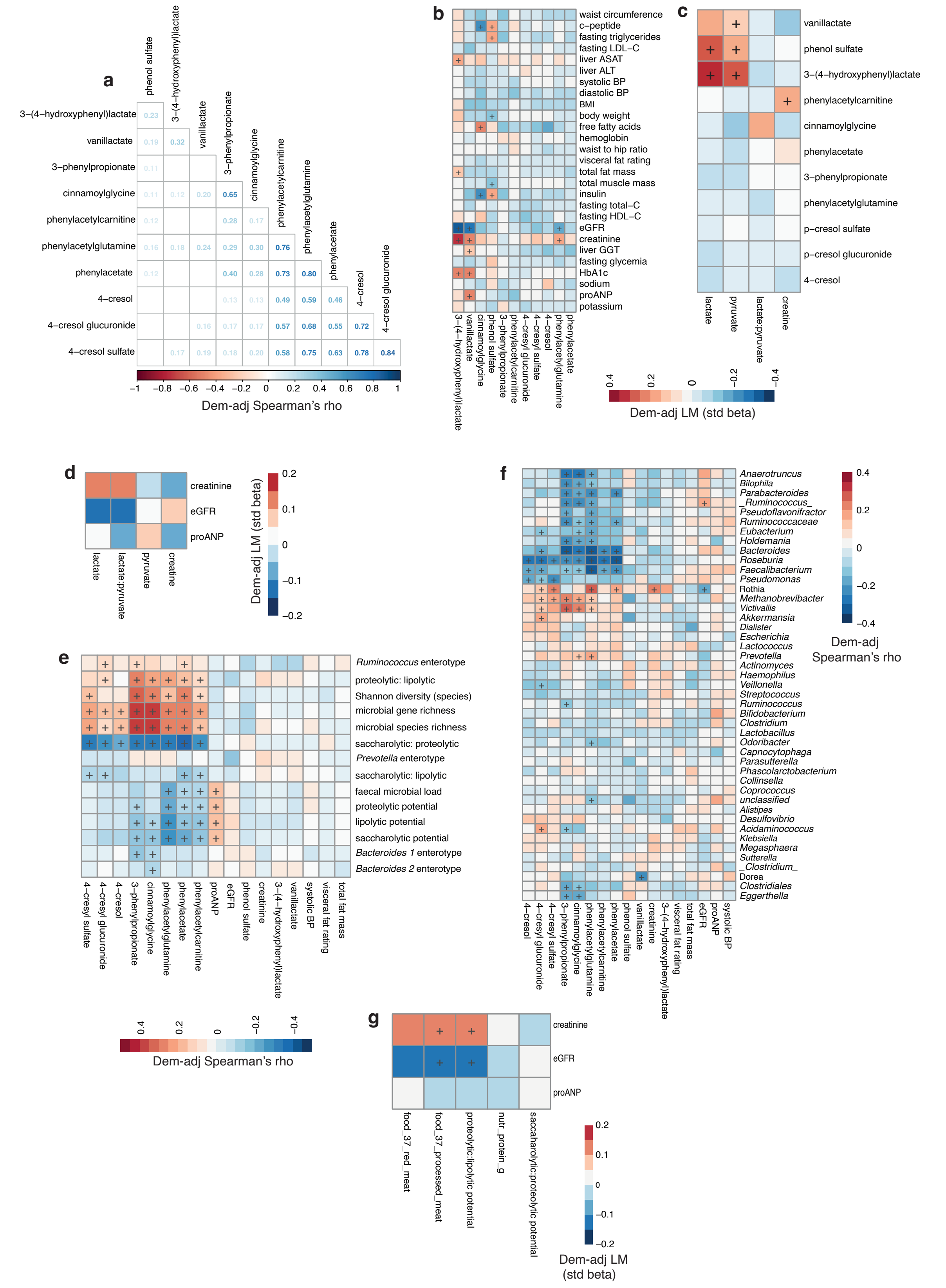

Supplementary Fig. 9. Microbial metabolites derived from phenylalanine and tyrosine metabolism associate with gut microbial features and bioclinical variables in healthy individuals. a, Correlogram representing demographics-adjusted two-sided partial Spearman

rank correlations among key metabolites derived from phenylalanine and tyrosine metabolism where the number within the box depicts Spearman's rho. Only correlations exhibiting FDR < 0.1 are shown with the color gradient following the intensity of the correlations (n = 247). Heatmaps representing associations of key metabolites with **b**, clinical variables (n = 230-273) and **c,** circulating markers of mitochondrial function in healthy individuals (n = 247-275). **d**, Heatmap representing the association between mitochondrial function markers and kidney-heart-related variables in healthy individuals (n = 254-272). Heatmaps representing associations of key metabolites with **e**, broader microbial features and **f,** microbial genera in healthy individuals (n = 248-275). For **e** and **f** clinical variables exhibiting significant associations with proANP (Supplementary Fig. 8a) were additionally included in the heatmaps. Heatmaps representing associations among **g,** key dietary and microbial features with kidney-heart variables in healthy individuals. Effect sizes (Spearman's rho for demographics-adjusted correlations; standardized beta for demographics-adjusted multiple linear regression using rank normalized data) and significance level of the tested variables are reported (n = 238-273; multiple testing corrections was done using BH method and FDR < 0.1 was considered significant). Source data are provided as a Source Data file.

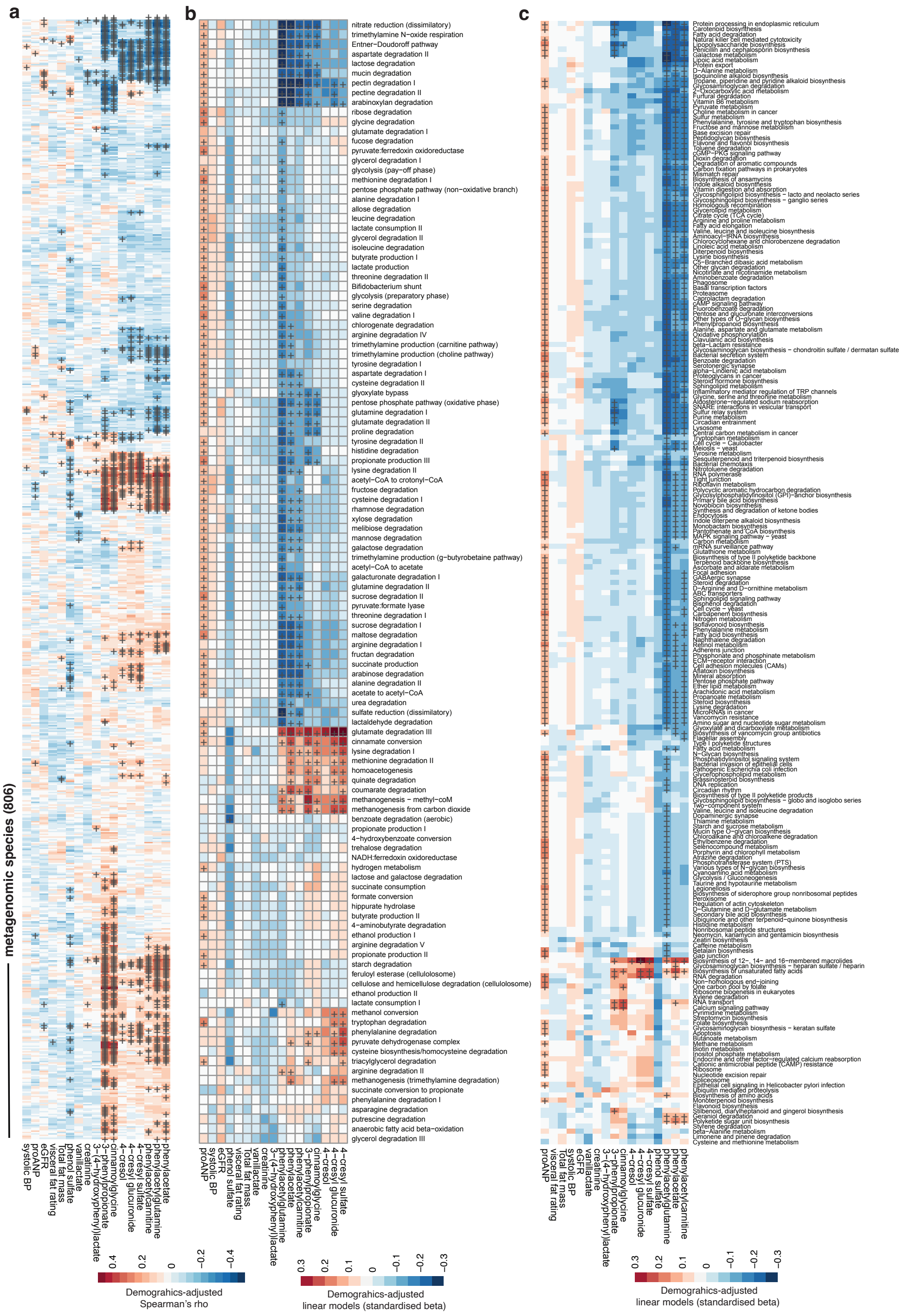

Supplementary Fig. 10. Microbial metabolites derived from phenylalanine and tyrosine metabolism associate with gut microbial compositional and functional features in healthy individuals. Heatmaps representing associations of key metabolites with **a**, MGS, **b,** GMM and, **c,** KEGG pathway in the MetaCardis healthy individuals (n = 247-275). Clinical variables exhibiting significant associations with proANP were additionally included in the heatmaps (Supplementary Fig. 8a). MGS-metabolite associations are further detailed in Supplementary Data 11. Effect sizes (Spearman's rho for demographics-adjusted correlations; standardized beta for demographics-adjusted multiple linear regression using rank normalized data) and significance level of the tested variables are reported (n = 248-275; multiple testing corrections was done using BH method and FDR < 0.1 was considered significant. MGS, metagenomic species, GMM, gut metabolic modules, BH, Benjamini-Hochberg; FDR, false-discovery rate. Source data are provided as a Source Data file.

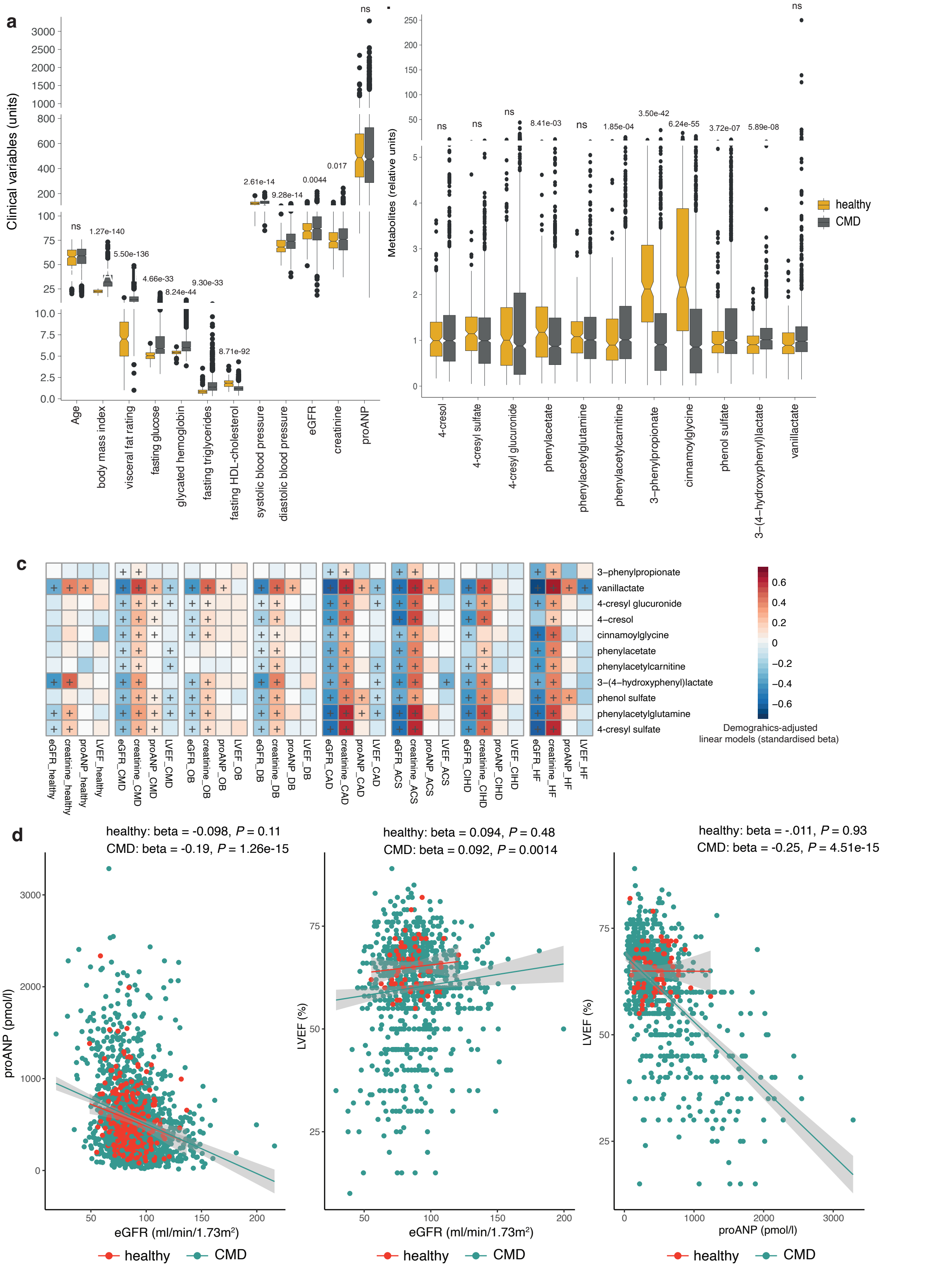

Supplementary Fig. 11. **Clinical variables and key metabolites in the MetaCardis healthy and CMD participants.** Box plots comparing distributions of key **a**, CMD-related clinical variables and **b**, metabolites derived from phenylalanine and tyrosine metabolism in healthy (n = 248-275) and CMD individuals (n = 1497-1602) of the MetaCardis study. The body of the

boxplot represents the first and third quartiles of the distribution with the median line, and the whiskers extend from the quartiles to the last data point within 1.5× interquartile range with outliers beyond. *P*-values were derived from demographics-adjusted ANCOVA models. **c**, Heatmaps showing associations among kidney-heart variables including LVEF and key metabolites derived from phenylalanine and tyrosine metabolism in the MetaCardis population segregated for disease groups (i.e., OB, overweight and obese, n = 682; DB, diabetics, n = 552; IHD, people with IHD, n = 368. The IHD group further included cases of ACS, n = 111; CHID, n =159; and HF due to CIHD, n = 98). Effect sizes represent demographics-adjusted multiple linear regression using rank normalized data; multiple testing corrections done using BH method; + represents significance as FDR < 0.1. **d,** Scatter plots showing relationships among eGFR, circulating proANP levels and LVEF in the MetaCardis healthy and CMD individuals tested using demographics-adjusted linear models using rank-normalized data. LVEF was only available in 72 healthy individuals from France and 850 CMD individuals. As a result, LVEF models in healthy individuals only included adjustment for age and sex. IHD, ischemic heart disease; CIHD, chronic IHD; HF, heart failure; LVEF, left ventricular ejection fraction. Source data are provided as a Source Data file.

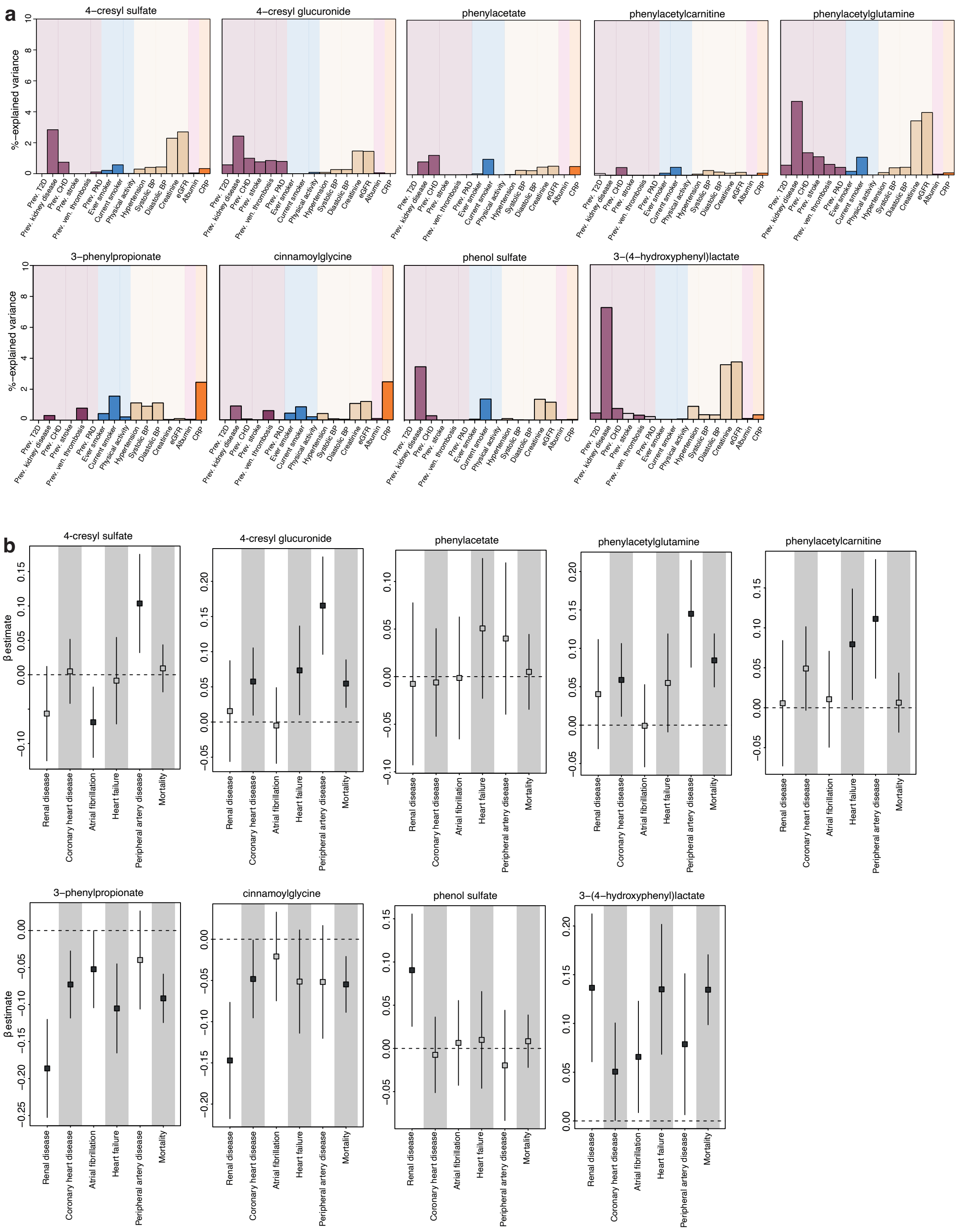

Supplementary Fig. 12. Determinants of key metabolites derived from phenylalanine and tyrosine metabolism and their respective associations with incident CVD in the EPIC-Norfolk cohort. a, Percent variance explained in plasma levels of key metabolites by selected risk factors derived from the EPIC-Norfolk cohort (n = 11,966) as described in Pietzner et al., 2021[35]. Plots were downloaded from their open-access web server (https://omicscience.org/apps/mwasdisease/). Briefly, Pietzner et al. assessed relevance of clinical risk factors and 50 traits measured at baseline in two ways: (1) using linear regression models to test for an association between traits as exposure and metabolite levels as outcome adjusting for age, sex, fasting time and time of blood sampling; and (2) variance in metabolite levels explained by each trait using variance partitioning as implemented in the R package

variancePartition (v1.14.1). **b,** Cox proportional hazards models derived estimates for associations of metabolite levels (log-transformed and standardized) with incident disease in the EPIC-Norfolk cohort (n = 11,966), with age as the underlying timescale adjusting for sex, as described in Pietzner et al., 2021[35]. Plots were downloaded from their open-access web server (https:// omicscience.org/apps/mwasdisease/). Filled squares represent significance at FDR < 0.1 for six tested diseases per metabolite where multiple testing corrections using BH method were applied to p-values derived from meta-analyses (pval.meta) downloaded from their server.

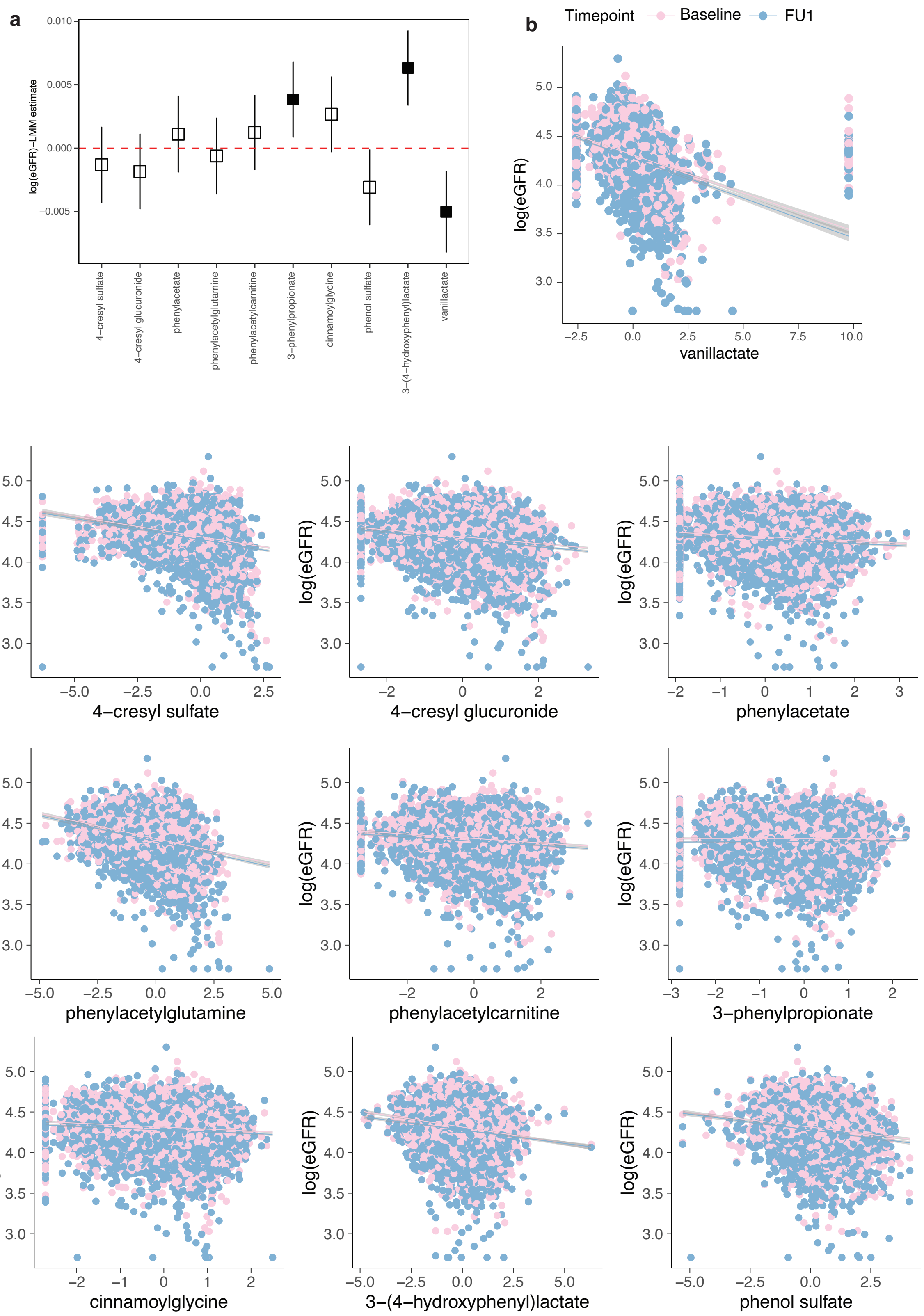

Supplementary Fig. 13. Associations among key metabolites derived from phenylalanine and tyrosine metabolism and kidney function in longitudinal settings in the CLSA study. a, Forest plot representing the relationships among key metabolites measured at baseline and change in eGFR in the CLSA study over the 3-year FU1 period (n

= 8,669). Estimates represent the interaction terms of linear mixed models using metabolites (log-transformed and standardized) as exposures and log eGFR as outcome with age, sex and time (days since baseline) as covariates involving an interaction term with the timepoints. Filled squares represent significance at FDR < 0.1 for ten tested metabolites (4-cresol was not available in CLSA). **b**, Scatter plots showing relationships among eGFR and key metabolites at two time points (i.e., baseline and FU1) in the CLSA study. Source data are provided as a Source Data file.

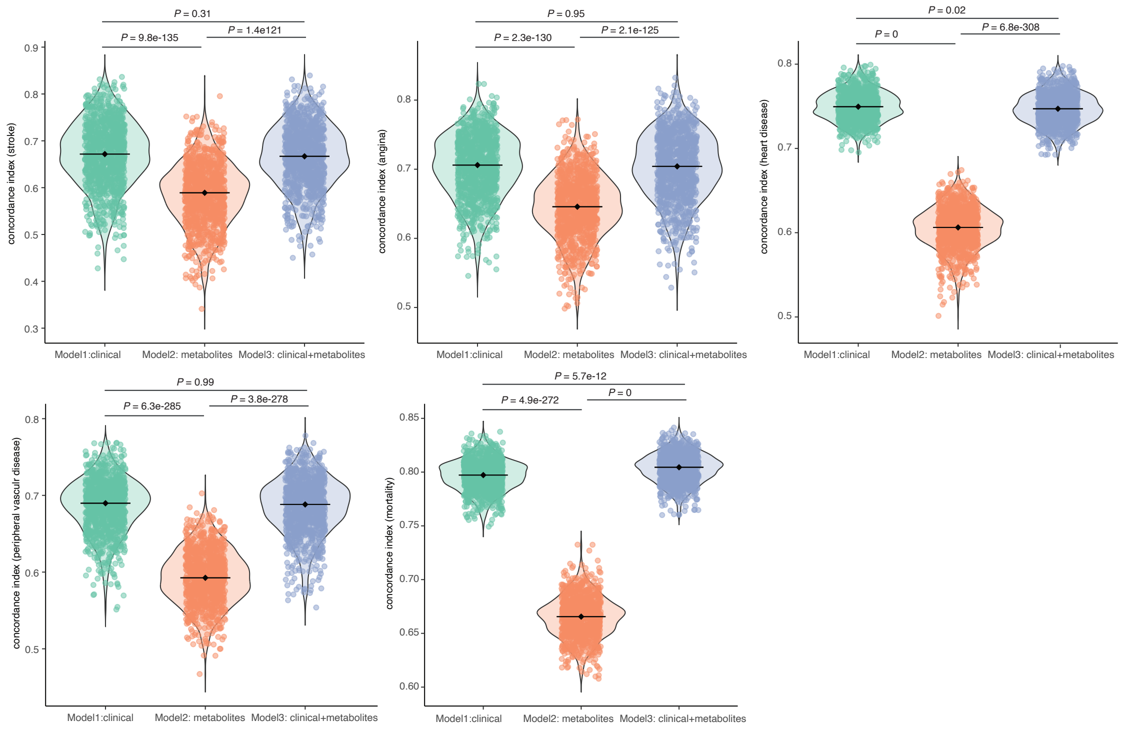

Supplementary Fig. 14: **Predictive analyses of key metabolites and clinical variables in the CLSA study.** Model 1 included baseline clinical variables relevant to heart disease (i.e., age, sex, body mass index, systolic blood pressure, glycated hemoglobin (factored as >5.7, 5.7–6.4 and <6.4 mmol/L), smoking status, fasting LDL- cholesterol, fasting triglycerides and HDL-cholesterol (mmol l) and eGFR) as predictors. Model 2 included six key metabolites with MR evidence (i.e., phenylacetylcarnitine, cinnamoylglycine, 4-cresyl sulfate, phenylacetylglutamine, vanillactate and 3-(4-hydroxyphenyl)lactate) and model 3 included both clinical and metabolites as predictors. Predictions were made using models randomly splitting the CLSA population with FU1 data into training (70%) and test (30%) sets, followed by five-fold cross-validations per training set and testing using the held-out test set, with 1000 bootstraps. *P*-values were derived from Kruskal-Wallis test with Dunn-Bonferroni *post hoc* tests. eGFR, estimated glomerular filtration ratio calculated according to MDRD formula. Source data are provided as a Source Data file.

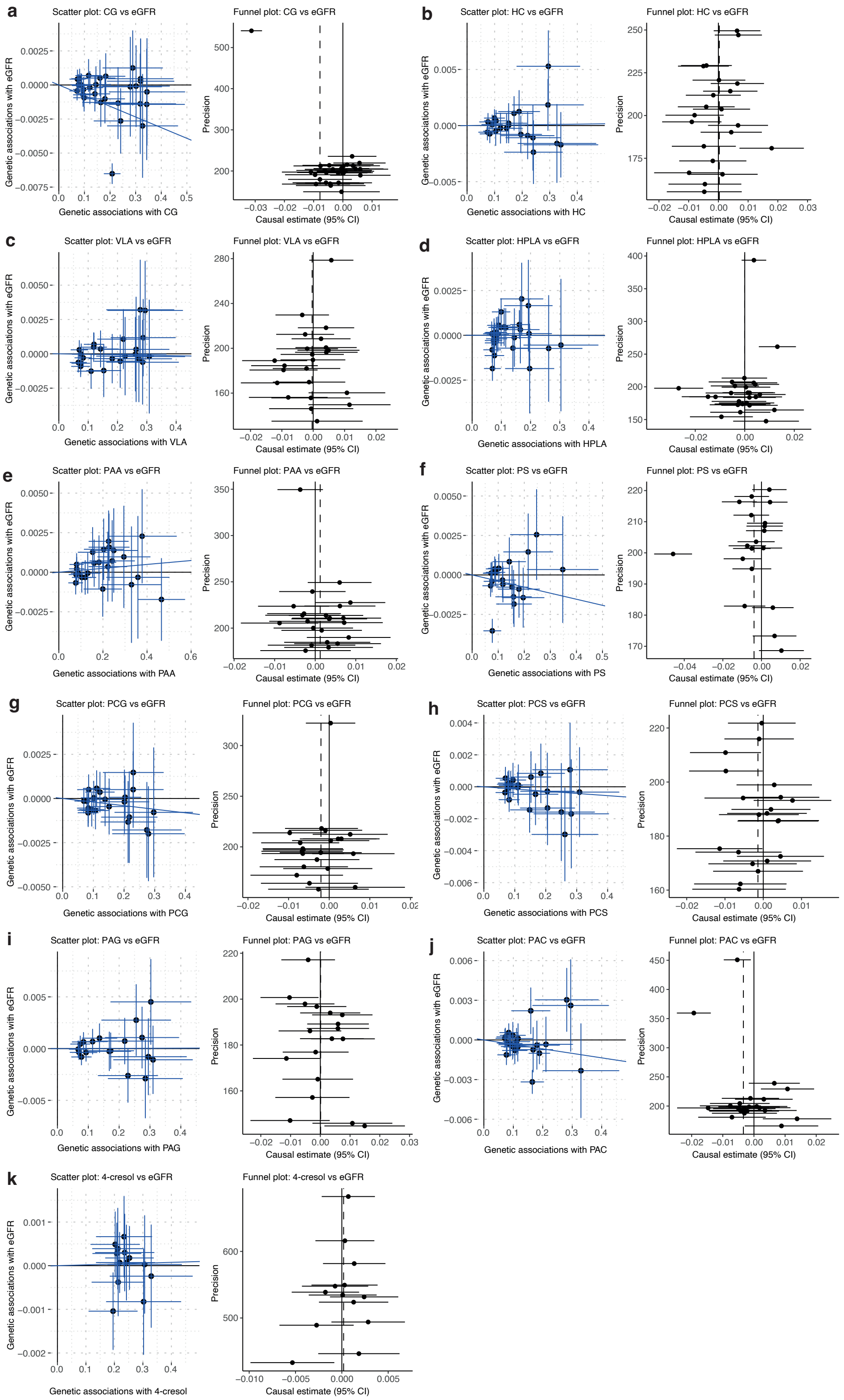

Supplementary Fig. 15. Univariate Mendelian Randomization analyses of circulating metabolites on kidney function. Scatter and funnel plots revealing the associations among genetic variants associated with individual key metabolites of the phenylalanine-tyrosine pathway as exposures and eGFR as an outcome

(Supplementary Data 17). Genetic associations for eGFR were derived from CKDGen and for metabolites from CLSA and EGEA (please see methods). PCS, 4-cresyl sulfate; PCG, 4-cresyl glucuronide; PAA,phenylacetate; PAG, phenylacetylglutamine; PAC, phenylacetylcarnitine; HC, 3-phenylpropionate; CG, cinnamoylglycine; PS, phenol sulfate; HPLA, 3-(4-hydroxyphenyl)-lactate; VLA, vanillactate; proANP, pro-atrial natriuretic peptide; eGFR, estimated glomerular filtration ratio calculated using CKDepi-creatinine equation.

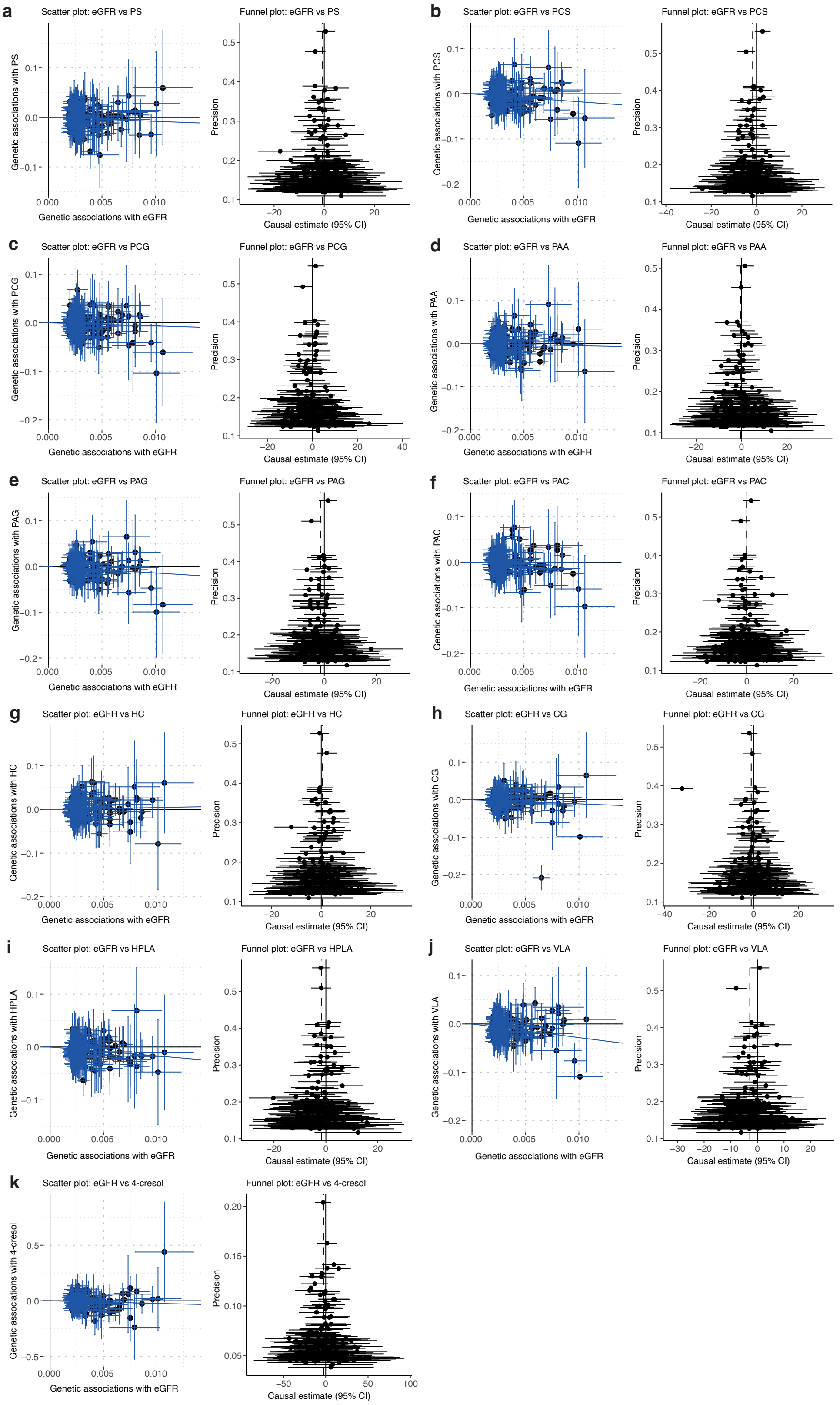

Supplementary Fig. 16. Univariate Mendelian Randomization analyses of kidney function on circulating metabolites. Scatter and funnel plots revealing the associations among genetic variants associated with eGFR as an exposure and key

metabolites of the phenylalanine-tyrosine pathway as outcomes (Supplementary Data 18). Genetic associations for eGFR were derived from CKDGen and for metabolites from CLSA and EGEA (please see methods). PCS, 4-cresyl sulfate; PCG, 4-cresyl glucuronide; PAA, phenylacetate; PAG, phenylacetylglutamine; PAC, phenylacetylcarnitine; HC, 3-phenylpropionate; CG, cinnamoylglycine; PS, phenol sulfate; HPLA, 3-(4-hydroxyphenyl)-lactate; VLA, vanillactate; proANP, pro-atrial natriuretic peptide; eGFR, estimated glomerular filtration ratio calculated using CKDepi-creatinine equation.

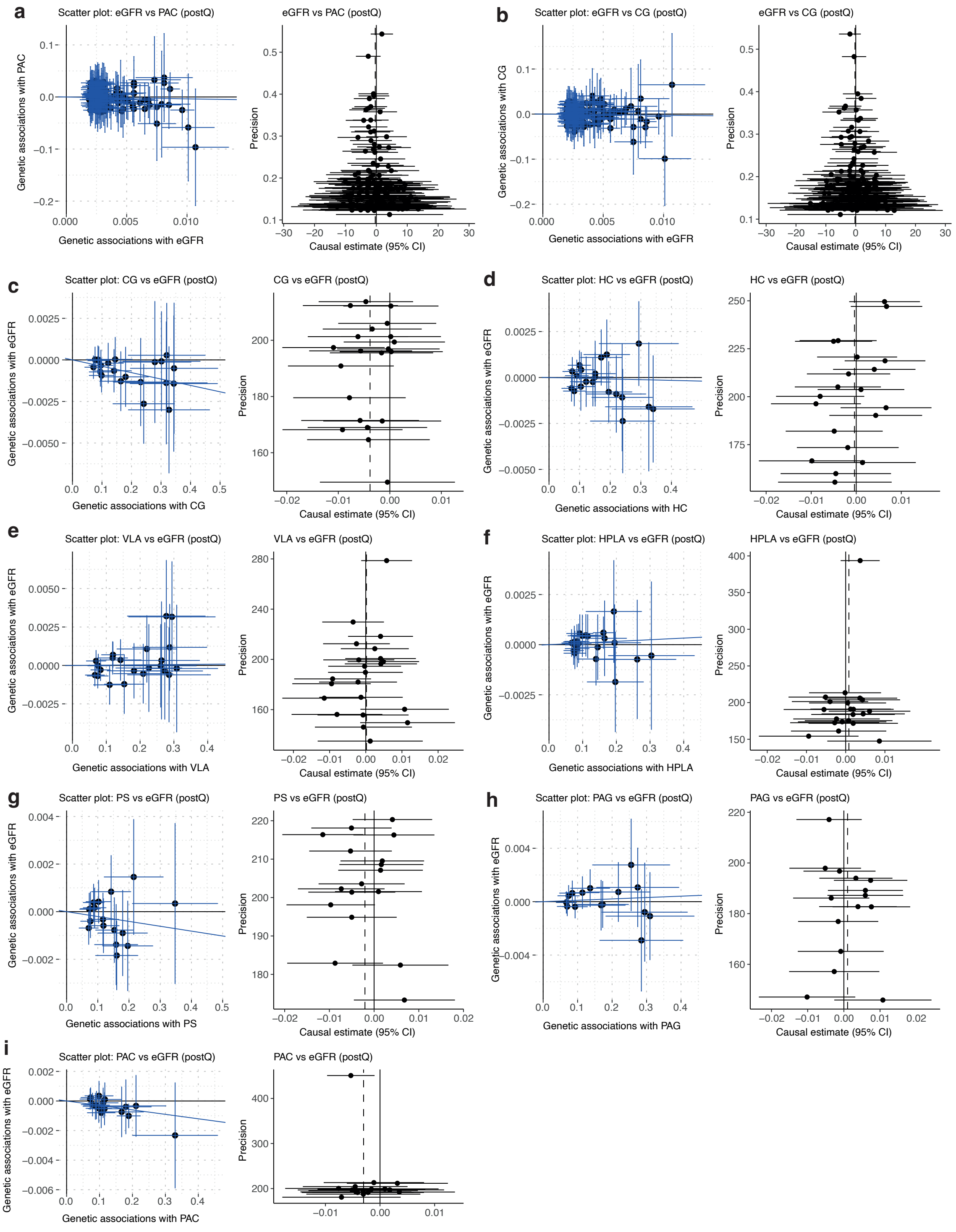

Supplementary Fig. 17. Bi-directional univariate Mendelian Randomization analyses of circulating metabolites on kidney function post outlier correction. Scatter and funnel plots revealing the associations among genetic variants associated with individual key metabolites and eGFR post Cochrane's Q based outlier removal. Genetic associations for eGFR were derived from CKDGen and for metabolites from CLSA and EGEA (please see methods). Only associations exhibiting significant heterogeneity (as measured by Cochrane's $Q \leq 0.05$ and I^2 statistics ≥ 25%) subjected to Q-based outlier corrections are shown here (Supplementary Data 17 and 18). PCS,

4-cresyl sulfate; PCG, 4-cresyl glucuronide; PAA, phenylacetate; PAG, phenylacetylglutamine; PAC, phenylacetylcarnitine; HC, 3-phenylpropionate; CG, cinnamoylglycine; PS, phenol sulfate; HPLA, 3-(4-hydroxyphenyl)-lactate; VLA, vanillactate; proANP, pro-atrial natriuretic peptide; eGFR, estimated glomerular filtration ratio calculated using CKDepi-creatinine equation.

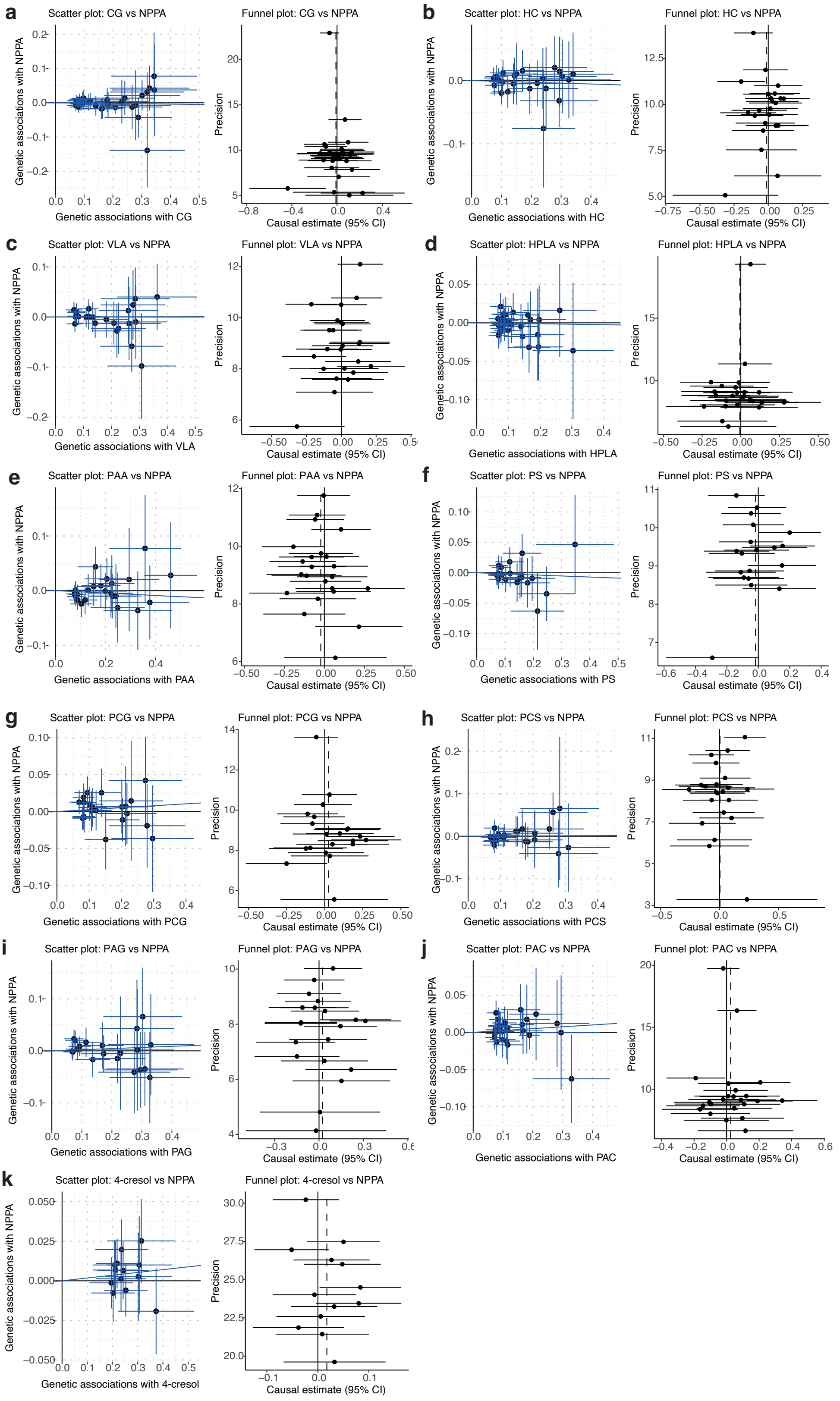

Supplementary Fig. 18. Univariate Mendelian Randomization analyses of circulating metabolites on NPPA. Scatter and funnel plots revealing the associations among genetic variants associated with individual key metabolites of the

phenylalanine-tyrosine pathway as exposures and NPPA as an outcome (Supplementary Data 19). Genetic associations for NPPA were derived from deCODE and for metabolites from CLSA and EGEA (please see methods). PCS, 4-cresyl sulfate; PCG, 4-cresyl glucuronide; PAA, phenylacetate; PAG, phenylacetylglutamine; PAC, phenylacetylcarnitine; HC, 3-phenylpropionate; CG, cinnamoylglycine; PS, phenol sulfate; HPLA, 3-(4-hydroxyphenyl)-lactate; VLA, vanillactate; NPPA, Somascan based natriuretic peptide A relative quantification.

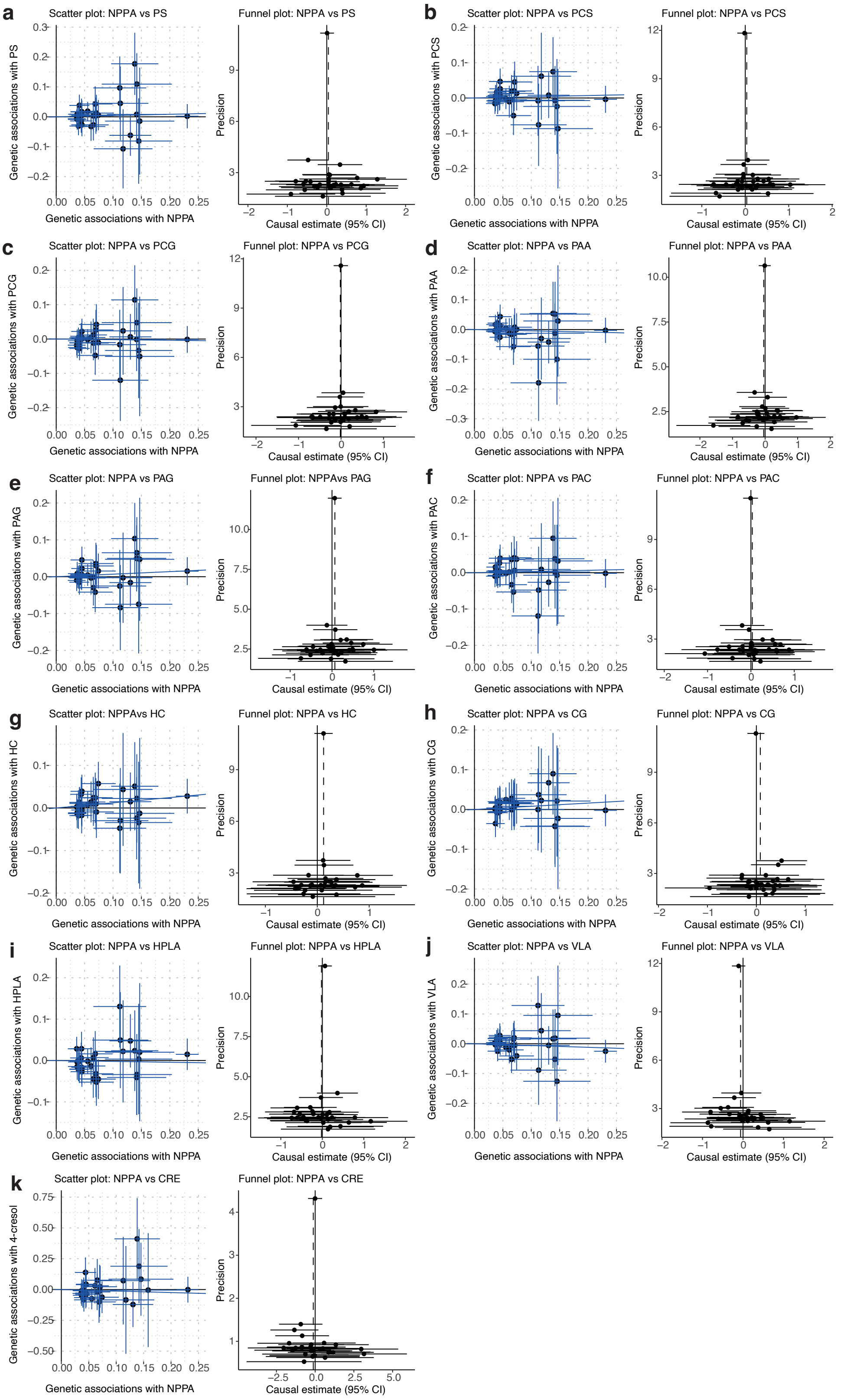

Supplementary Fig. 19. Univariate Mendelian Randomization analyses of NPPA on circulating metabolites. Scatter and funnel plots revealing the associations among genetic variants associated with NPPA as exposure and key metabolites of the

phenylalanine-tyrosine pathway as outcomes (Supplementary Data 20). Genetic associations for NPPA were derived from deCODE and for metabolites from CLSA and EGEA (please see methods). PCS, 4-cresyl sulfate; PCG, 4-cresyl glucuronide; PAA, phenylacetate; PAG, phenylacetylglutamine; PAC, phenylacetylcarnitine; HC, 3-phenylpropionate; CG, cinnamoylglycine; PS, phenol sulfate; HPLA, 3-(4-hydroxyphenyl)-lactate; VLA, vanillactate; NPPA, Somascan based natriuretic peptide A relative quantification.

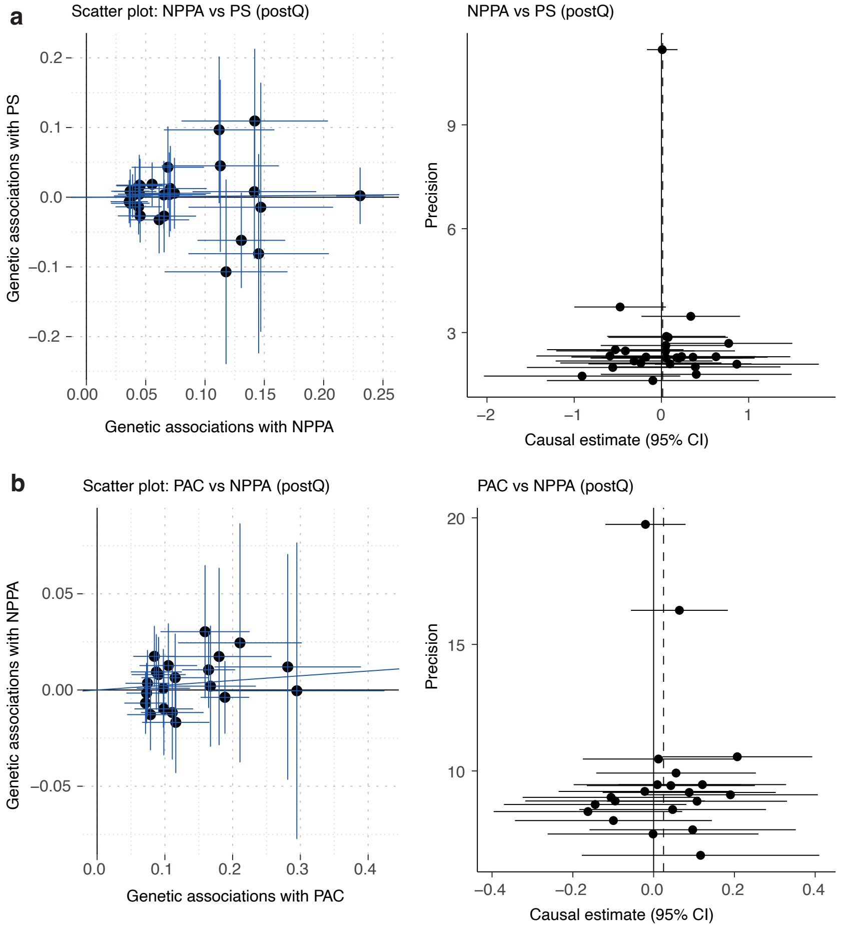

Supplementary Fig. 20. Bi-directional univariate Mendelian Randomization analyses of circulating metabolites on NPPA post outlier correction. Scatter and funnel plots revealing the associations among genetic variants associated with individual key metabolites and NPPA post Cochrane's Q based outlier removal. Genetic associations for NPPA were derived from deCODE and for metabolites from CLSA and EGEA (please see methods). Only associations exhibiting significant heterogeneity (as measured by Cochrane's Q≤0.05 and I^2 statistics ≥ 25%) subjected to Q-based outlier corrections are shown here (Supplementary Data 19 and 20). PCS, 4-cresyl sulfate; PCG, 4-cresyl glucuronide; PAA, phenylacetate; PAG, phenylacetylglutamine; PAC, phenylacetylcarnitine; HC, 3-phenylpropionate; CG, cinnamoylglycine; PS, phenol sulfate; HPLA, 3-(4-hydroxyphenyl)-lactate; VLA, vanillactate; NPPA, Somascan based natriuretic peptide A relative quantification.

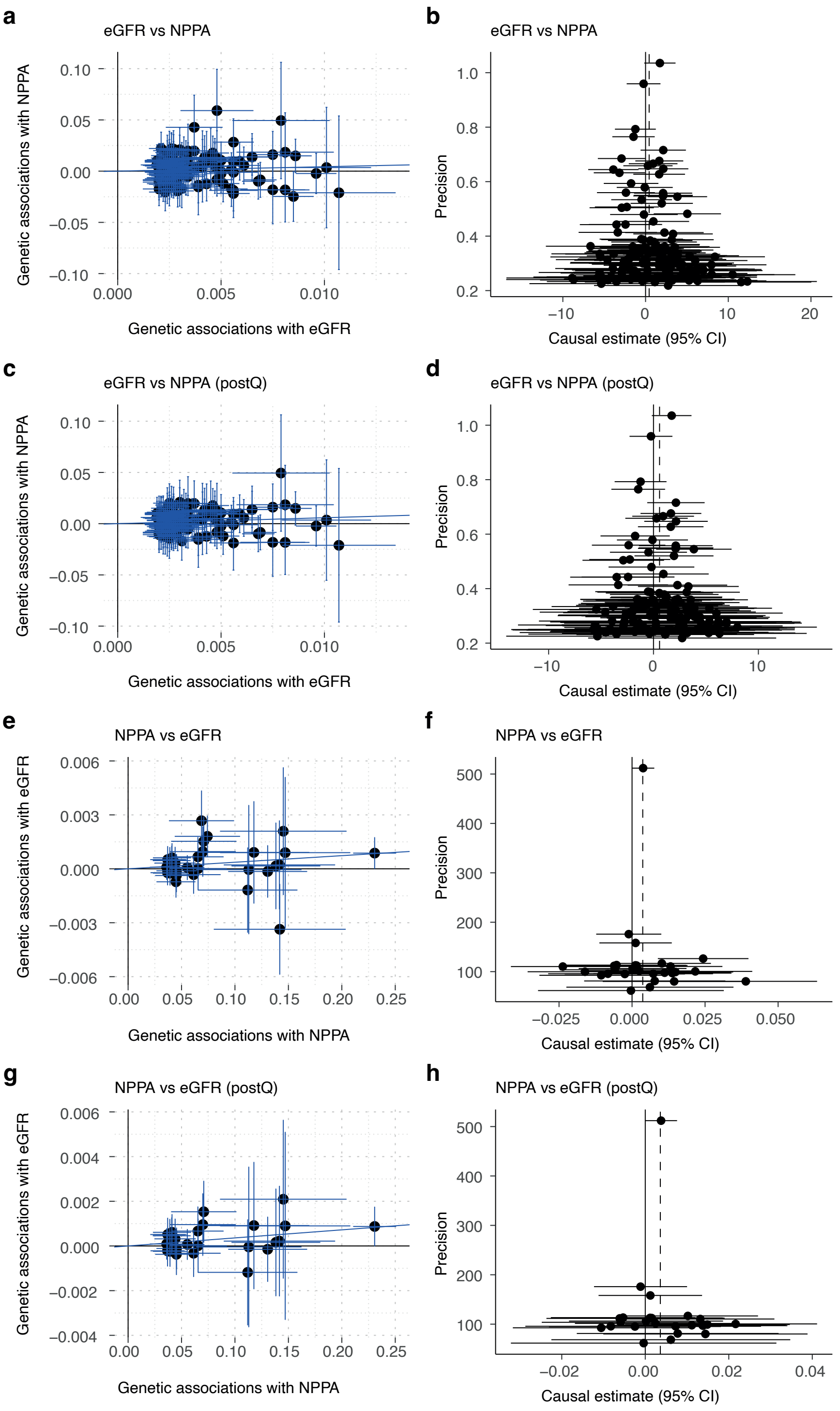

Supplementary Figure 21. Bi-directional univariate Mendelian Randomization analyses of eGFR-NPPA axis. Scatter and funnel plots revealing the associations among genetic variants associated with eGFR and NPPA pre- and post- Cochrane's Q based outlier removal (Supplementary Data 21). Genetic associations for eGFR and NPPA were derived from CKDGen and deCODE, respectively (please see methods). eGFR, estimated glomerular filtration ratio calculated using CKDepi-creatinine equation; NPPA, Somascan based natriuretic peptide A relative quantification.

Supplemental Table 1: The STORMS checklist.

Number	Item	Recommendation	Item Source	Additional Guidance	Yes/No/NA	
**Abstract**						
1.0	Structured or Unstructured Abstract	Abstract should include information on background, methods, results, and conclusions in structured or unstructured format.	STORMS		Yes	
1.1	Study Design	State study design in abstract.	STORMS	See 3.0 for additional information on study design.	No	
1.2	Sequencing methods	State the strategy used for metagenomic classification.	STORMS	For example, targeted 16S by qPCR or sequencing, shotgun metagenomics, metatranscriptomics, etc.	No	
1.3	Specimens	Describe body site(s) studied.	STORMS		Yes	
**Introduction**						
2.0	Background and Rationale	Summarize the underlying background, scientific evidence, or theory driving the current hypothesis as well as the study objectives.	STORMS		Yes	
2.1	Hypotheses	State the pre-specified hypothesis. If the study is exploratory, state any pre-specified study objectives.	STORMS		Yes	
**Methods**						
3.0	Study Design	Describe the study design.	STORMS	Observational (Case-Control, Cohort, Cross-sectional survey, etc.) or Experimental (Randomized controlled trial, Non-randomized controlled trial, etc.). For a brief description of common study designs see: DOI: 10.11613/BM.2014.022	Observational study in case-control settings	

				If applicable, describe any blinding (e.g. single or double-blinding) used in the course of the study.		
3.1	Participants	State what the population of interest is, and the method by which participants are sampled from that population. Include relevant information on physiological state of the subjects or stage in the life history of disease under study when participants were sampled.	STORMS	Examples of the population of interest could be: adults with no chronic health conditions, adults with type II diabetes, newborns, etc. This is the total population to whom the study is hoped to be generalizable to. The sampling method describes how potential participants were selected from that population.<br><br>If the participants are from a substudy of a larger study, provide a brief description of that study and cite that study.<br><br>Clearly state how cases and controls are defined.<br><br>An example of relevant physiological state might be pre/post menopausal for a vaginal microbiome study; examples of stage in the life history of disease could be whether specimens were collected during active or dormant disease, or before or after treatment.	Yes	
3.2	Geographic location	State the geographic region(s) where participants were sampled from.	MIxS: geographic location (country	Geographic coordinates can be reported to prevent potential ambiguities if necessary.	Yes	

			and/or sea,region)			
3.3	Relevant Dates	State the start and end dates for recruitment, follow-up, and data collection.	STORMS	Recruitment is the period in which participants are recruited for the study. In longitudinal studies, follow-up is the date range in which participants are asked to complete a specific assessment. Finally, data collection is the total period in which data is being collected from participants including during initial recruitment through all follow-ups.	Yes	
3.4	Eligibility criteria	List any criteria for inclusion and exclusion of recruited participants.	Modified STROBE	Among potential recruited participants, how were some chosen and others not? This could include criteria such as sex, diet, age, health status, or BMI.<br><br>If there is a primary and validation sample, describe inclusion/exclusion criteria for each.	Yes	
3.5	Antibiotics Usage	List what is known about antibiotics usage before or during sample collection.	STORMS	If participants were excluded due to current or recent antibiotics usage, state this here.<br><br>Other factors (e.g. proton pump inhibitors, probiotics, etc.) that may influence the microbiome should also be described as well.	Yes	

3.6	Analytic sample size	Explain how the final analytic sample size was calculated, including the number of cases and controls if relevant, and reasons for dropout at each stage of the study. This should include the number of individuals in whom microbiome sequencing was attempted and the number in whom microbiome sequencing was successful.	STORMS	Consider use of a flow diagram (see template at https://stormsmicrobiome.org/figures) . Also state sample size in abstract.<br><br>If power analysis was used to calculate sample size, describe those calculations.	All samples included in the current work had sequencing data available.	
3.7	Longitudinal Studies	For longitudinal studies, state how many follow-ups were conducted, describe sample size at follow-up by group or condition, and discuss any loss to follow-up.	STORMS	If there is loss to follow-up, discuss the likelihood that drop-out is associated with exposures, treatments, or outcomes of interest.	N/A	
3.8	Matching	For matched studies, give matching criteria.	Modified STROBE	"Matched" refers to matching between comparable study participants as cases and controls or exposed / unexposed.<br><br>Indicate whether participants were individual or frequency matched and in what ratio were they matched (e.g. 1 case to 1 control).	N/A	
3.9	Ethics	State the name of the institutional review board that approved the study and protocols, protocol number and date of approval, and procedures for obtaining informed consent from participants.	STORMS		Yes	
4.0	Laboratory methods	State the laboratory/center where laboratory work was done.	STORMS	Provide a reference to complete lab protocols if previously published elsewhere such as on protocols.io. Note any modifications of lab protocols and the reason for protocol modifications.	Yes	

4.1	Specimen collection	State the body site(s) sampled from and how specimens were collected.	MIxS: sample collection device or method; host body site	Use terms from the Uber-anatomy Ontology (https://www.ebi.ac.uk/ols/ontologies/uberon) to describe body sites in a standardized format.	Yes	
4.2	Shipping	Describe how samples were stored and shipped to the laboratory.	STORMS	Include length of time from collection to receipt by the lab and if temperature control was used during shipping.	Length of time from collection not available; temperature control during shipping implemented	
4.3	Storage	Describe how the laboratory stored samples, including time between collection and storage and any preservation buffers or refrigeration used.	STORMS	State where each procedure or lot of samples was done if not all in the same place.<br><br>Include reagent/lot/catalogue #s for storage buffers.	Yes	
4.4	DNA extraction	Provide DNA extraction method, including kit and version if relevant.	MIxS: nucleic acid extraction	If any DNA quantification methods were used prior to DNA amplification or at the pooling step of library preparation, state so here.	Total faecal DNA was extracted following the IHMS guidelines (SOP-07V2 H).	
4.5	Human DNA sequence depletion or microbial DNA enrichment	Describe whether human DNA sequence depletion or enrichment of microbial or viral DNA was performed.	STORMS		Cleaned reads were subsequently filtered from human and other possible food contaminant DNA (using Human genome RCh37-p10, *Bos taurus* and *Arabidopsis thaliana* with an identity score threshold of 97%).	

4.6	Primer selection	Provide primer selection and DNA amplification methods as well as variable region sequenced (if applicable).	MIxS: pcr primers		N/A	
4.7	Positive Controls	Describe any positive controls (mock communities) if used.	STORMS	If used, should be deposited under guidance provided in the 8.X items.	Not used	
4.8	Negative Controls	Describe any negative controls if used.	STORMS	If used, should be deposited under guidance provided in the 8.X items.	Not used	
4.9	Contaminant mitigation and identification	Provide any laboratory or computational methods used to control for or identify microbiome contamination from the environment, reagents, or laboratory.	STORMS	Includes filtering of reagents and other steps to minimize contamination. It is relevant to state whether the specimens of interest have low microbial load, which makes contamination especially relevant.	None specifically	
4.10	Replication	Describe any biological or technical replicates included in the sequencing, including which steps were replicated between them.	STORMS	Replication may be biological (redundant biological specimens) or technical (aliquots taken at different stages of analysis) and used in extraction, sequencing, preprocessing, and/or data analysis.	No replication, any technical bias was handled using rarefaction of read counts.	
4.11	Sequencing strategy	Major divisions of strategy, such as shotgun or amplicon sequencing.	MIxS: sequencing method	For amplicon sequencing (for example, 16S variable region), state the region selected. State the model of sequencer used.	Shotgun sequencing	
4.12	Sequencing methods	State whether experimental quantification was used (QMP/cell count based, spike-in based) or whether relative abundance methods were applied.	STORMS	These include read length, sequencing depth per sample (average and minimum), whether reads are paired, and other parameters.	QMP-based quantification.	
4.13	Batch effects	Detail any blocking or randomization used in study design to avoid confounding of batches with	STORMS	Sources of batch effects include sample collection, storage, library preparation, and sequencing and are	No significant batch effects were reported.	

		exposures or outcomes. Discuss any likely sources of batch effects, if known.		commonly unavoidable in all but the smallest of studies.		
4.14	Metatranscriptomics	Detail whether any mRNA enrichment was performed and whether/how retrotranscription was performed prior to sequencing. Provide size range of isolated transcripts. Describe whether the sequencing library was stranded or not. Provide details on sequencing methods and platforms.	STORMS	Provide details on any internal standards which may have been used as well as parameters and versions of any software or databases used.	N/A	
4.15	Metaproteomics	Detail which protease was used for digestion. Provide details on proteomic methods and platforms (e.g. LC-MS/MS, instrument type, column type, mass range, resolution, scan speed, maximum injection time, isolation window, normalised collision energy, and resolution).	STORMS	Provide details on any internal standards which may have been used as well as parameters and versions of any software or databases used.	N/A	
4.16	Metabolomics	Specify the analytic method used (such as nuclear magnetic resonance spectroscopy or mass spectrometry). For mass spectrometry, detail which fractions were obtained (polar and/or non-polar) and how these were analyzed. Provide details on metabolomics methods and platforms (e.g. derivatization, instrument type, injection type, column type and instrument settings).	STORMS	Provide details on any internal standards which may have been used as well as parameters and versions of any software or databases used.	Yes	
5.0	Data sources/ measurement	For each non-microbiome variable, including the health condition, intervention, or other variable of interest, state how it was defined, how it was measured or collected, and any transformations applied to the variable prior to analysis.	MIxS: host disease status	State any sources of potential bias in measurements, for example multiple interviewers or measurement instruments, and whether these potential biases were assessed or accounted for in study design.<br><br>Use terms from a standardized ontology such as the Experimental Factor Ontology	Biases due to host variables were explicitly tested and accounted for.	

				(https://www.ebi.ac.uk/efo/) to describe variables of interest in a standardized format.		
6.0	Research design for causal inference	Discuss any potential for confounding by variables that may influence both the outcome and exposure of interest. State any variables controlled for and the rationale for controlling for them.	STORMS	For causal inference, this item refers to describing the assumptions that would be required to draw causal inferences from observational data. See Vujkovic-Cvijin, I., Sklar, J., Jiang, L. et al. Host variables confound gut microbiota studies of human disease. Nature 587, 448–454 (2020). https://doi.org/10.1038/s41586-020-2881-9 for more details on confounding in observational microbiome studies.<br><br>For example, hypothesized confounders may be controlled for by multivariable adjustment. Consider using a directed acyclic graph (DAG) to describe your causal model and justify any variables controlled for. DAGs can be made using www.dagitty.net.	Yes, we have dealt with this by explicit testing and accounting for key confounders.	

6.1	Selection bias	Discuss potential for selection or survival bias.	STORMS	Selection bias can occur when some members of the target study population are more likely to be included in the study/final analytic sample than others. Some examples include survival bias (where part of the target study population is more likely to die before they can be studied), convenience sampling (where members of the target study population are not selected at random), and loss to follow-up (when probability of dropping out is related to one of the things being studied).	Selection bias is likely as the recruitment of study subjects was done to derive a population exhibiting a range of cardiometabolic disease spectrum. Owing to the nature of the study design, individuals were recruited from clinical settings and not randomly from the general population.	
7.0	Bioinformatic and Statistical Methods	Describe any transformations to quantitative variables used in analyses (e.g. use of percentages instead of counts, normalization, rarefaction, categorization).	STORMS	If a variable is analyzed using different transformations, state rationale for the transformation and for each analyses which version of the variable is used.<br><br>In case of any complex or multistep transformations, give enumerated instructions for reproducing those transformations.	Described in the manuscript.	
7.1	Quality Control	Describe any methods to identify or filter low quality reads or samples.	MIxS: sequence quality check	If samples were excluded based on quality or read depth, list the criteria used, the number of samples excluded, and the final sample size after quality control.	All samples included in the current study passed the QC.	
7.2	Sequence analysis	Describe any taxonomic, functional profiling, or other sequence analysis performed.	MIxS: feature prediction; similarity		Described in the manuscript	

			search method			
7.3	Statistical methods	Describe all statistical methods.	Modified STROBE	Describe any statistical tests used, exploratory data analysis performed, dimension reduction methods/unsupervised analysis, alpha/beta metrics, and/or methods for adjusting for measurement bias.<br><br>If multiple statistical methods are possible, discuss why the methods used were selected.<br><br>If a multiple hypothesis testing correction method was used, describe the type of correction used.<br><br>State which taxonomic levels are analyzed.	Described in the manuscript	
7.4	Longitudinal analysis	If the study is longitudinal, include a section that explicitly states what analysis methods were used (if any) to account for grouping of measurements by individual or patterns over time.	STORMS		N/A	
7.5	Subgroup analysis	Describe any methods used to examine subgroups and interactions.	STROBE		N/A	
7.6	Missing data	Explain how missing data were addressed.	STROBE	"Missing data" refers to participant measurements such as covariates, exposures, outcomes, or time points that should have been collected but were not, not to zeros in taxonomic	Samples with missing data in any comparison were excluded unless otherwise specified.	

				abundance tables or data points not applicable to that observation.		
7.7	Sensitivity analyses	Describe any sensitivity analyses.	STROBE		None	
7.8	Findings	State criteria used to select findings for reporting.	STORMS	For example, false discovery rate with total number of tests, effect size threshold, significance threshold, microbes of interest.	Yes	
7.9	Software	Cite all software (including read mapping software) and databases (including any used for taxonomic reference or annotating amplicons, if applicable) used. Include version numbers.	Modified STREGA	Installed packages, add-ons or libraries should be stated and cited in addition to the software used.<br><br>All parameters employed that differ from the default of that software/version should be provided.<br><br>This is in addition to, not a replacement for, publishing of code as outlined in the section Reproducible Research.	Yes	

8.0	Reproducible research	Make a statement about whether and how others can reproduce the reported analysis.	STORMS	Any protected information that has been excluded or provided under controlled access should be listed along with any relevant data access procedures. "On request from authors" is not sufficiently detailed; formal data access procedures and conditions should be defined.<br><br>If data are unavailable, state so clearly.<br><br>Consider using a specialized rubric for reproducible research (such as: https://mbio.asm.org/content/9/3/e00525-18.short).<br><br>Consider preregistering the study protocol (such as on osf.io or https://plos.org/open-science/preregistration/).	A “Data Availability Statement” is provided.	
8.1	Raw data access	State where raw data may be accessed including demultiplexing information.	STORMS	Robust, long-term databases such as those hosted by NCBI and EBI are preferred. If using a private repository, provide rationale.	A “Data Availability Statement” is provided.	
8.2	Processed data access	State where processed data may be accessed.	STORMS	Unfiltered data should be provided.<br><br>Robust, long-term databases such as those hosted by NCBI and EBI-EMBL are preferred. Repositories like zenodo (https://zenodo.org/) or publisso (https://www.publisso.de/en/working-for-you/doi-service/)	A “Data Availability Statement” is provided.	

				can be used to provide a DOI and long-term storage for processed datasets, even those which cannot be published openly.		
8.3	Participant data access	State where individual participant data such as demographics and other covariates may be accessed, and how they can be matched to the microbiome data.	STORMS	If re-categorized, transformed, or otherwise derived variables were used in the analysis, these variables or code for deriving them should be provided.<br><br>Examples of how participant data can be matched to microbiome data are: using the same set of anonymized identifiers, or using different anonymized identifiers but providing a map.<br><br>Provided data should be sufficient to independently replicate the current analysis.	A “Data Availability Statement” is provided.	
8.4	Source code access	State where code may be accessed.	STORMS	If a standard or formalized workflow was employed, reference it here.	A “Code Availability Statement” is provided.	
8.5	Full results	Provide full results of all analyses, in computer-readable format, in supplementary materials.	STORMS	For example, any fold-changes, p-values, or FDR values calculated, provided as a spreadsheet.<br><br>Use a machine-readable, plain-text format such as csv or tsv.	Yes	
**Results**						

9.0	Descriptive data	Give characteristics of study participants (e.g. dietary, demographic, clinical, social) and information on exposures and potential confounders.	STROBE	Typically reported in a table included in the paper or as a supplementary table. Indicate number of participants with missing data for each variable of interest.<br><br>This includes environmental and lifestyle factors that may affect the relationship between the microbiome and the condition of interest. Participant diet and medication use should be summarized, if known.<br><br>At minimum, age and sex of all participants should be summarized.	Yes	
10.0	Microbiome data	Report descriptive findings for microbiome analyses with all applicable outcomes and covariates.	STORMS	This includes measures of diversity as well as relative abundances. These descriptive findings should be reported both for the sample overall and for individual groups.	Yes	
10.1	Taxonomy	Identify taxonomy using standardized taxon classifications that are sufficient to uniquely identify taxa.	STORMS	If not using full taxonomic hierarchy, make sure it is clear whether names stated are species, genera, family, etc.<br><br>Italicize genus/species pairs. Consult journal guidelines or standardized references on taxonomic nomenclature. For instance, https://wwwnc.cdc.gov/eid/page/scientific-nomenclature	Yes	

10.2	Differential abundance	Report results of differential abundance analysis by the variable of interest and (if applicable) by time, clearly indicating the direction of change and total number of taxa tested.	STORMS	If there are more than two groups, include omnibus (multigroup) test results if applicable to the research question.<br><br>If applicable, reported effect sizes should include a measure of uncertainty such as the confidence interval.	Yes	
10.3	Other data types	Report other data analyzed--e.g. metabolic function, functional potential, MAG assembly, and RNAseq.	STORMS		Yes	
10.4	Other statistical analysis	Report any statistical data analysis not covered above.	STORMS	This could include subgroup analysis, sensitivity analyses, and cluster analysis.<br><br>Visualizations should be easily interpretable and colorblind-friendly. The caption and/or main text should provide a detailed description of visualizations for visually-impaired readers.	Yes	
**Discussion**						
11.0	Key results	Summarise key results with reference to study objectives	STROBE		Yes	

12.0	Interpretation	Give a cautious overall interpretation of results considering objectives, limitations, multiplicity of analyses, results from similar studies, and other relevant evidence.	STROBE	Define or clarify any subjective terms such as "dominant," "dysbiosis," and similar words used in interpretation of results.<br><br>When interpreting the findings, consider how the interpretation of the findings may be summarized or quoted for the general public such as in press releases or news articles.<br><br>If causal language is used in the interpretation (such as "alters," "affects," "results in," "causes," or "impacts"), assumptions made for causal inference should be explicitly stated as part of 6.0 and 13.0.<br><br>Distinguish between function potential (ie inferred from metagenomics) and observed activity (ie metatranscriptomic, metabolomic, proteomic) if discussing microbial function.	Yes	
13.0	Limitations	Discuss limitations of the study, taking into account sources of potential bias or imprecision.	STROBE	Also consider limitations resulting from the methods (especially novel methods), the study design, and the sample size.	Yes	
13.1	Bias	Discuss any potential for bias to influence study findings.	STORMS	May include sampling method, representativeness of study participants, or potential confounding.	Yes	

13.2	Generalizability	Discuss the generalisability (external validity) of the study results	STROBE	To what populations or other settings do you expect the conclusions to generalize?	We have included a statement on generalizability in our discussion.	
14.0	Ongoing/future work	Describe potential future research or ongoing research based on the study's findings.	STORMS		Yes	
**Other information**						
15.0	Funding	Give the source of funding and the role of the funders for the present study and, if applicable, for the original study on which the present article is based	STROBE		Yes	
15.1	Acknowledgements	Include acknowledgements of those who contributed to the research but did not meet critera for authorship.	STORMS	For general guidelines on authorship, see http://www.icmje.org and https://www.elsevier.com/authors/journal-authors/policies-and-ethics/credit-author-statement	Yes	
15.2	Conflicts of Interest	Include a conflicts of interest statement.	STORMS		Yes	
16.0	Supplements	Indicate where supplements may be accessed and what materials they contain.	STORMS		Yes	
17.0	Supplementary data	Provide supplementary data files of results with for all taxa and all outcome variables analyzed. Indicate the taxonomic level of all taxa.	STORMS	Depending on the analysis performed, examples of the supplemental results included could be mean relative abundance, differential abundance, raw p-value, multiple hypothesis testing-adjusted p-values, and standard error.<br><br>All discussed taxa should include the taxonomic level (e.g. class, order, genus).	Yes	
