# Supplementary material for: A gut microbiome-kidney-heart axis predictive of future cardiovascular diseases
Source: Nat Commun. 2026 Mar 5;17:3477. doi: 10.1038/s41467-026-69405-0 (PMC13079829; doi:10.1038/s41467-026-69405-0)
Supplement: Supplementary file 2 — Description Of Additional Supplementary File [file 41467_2026_69405_MOESM2_ESM.pdf]

Description of Additional supplementary files

Supplementary data 1.

Characteristics of metabolically healthy controls (n = 275) and symptomatic subjects with known and diagnosed CVD (CMD individuals, n=1602) of the European MetaCardis study. CMD population (n = 1,602 comprised of individuals with metabolic syndrome, overweight and obesity (n = 682), T2D (n = 552) and IHD (n = 368). Median and first as well as third quartile limits are shown for non-normally distributed variables (median [q1; q3]). Group differences were estimated using Mann-Whitney U tests. Count data were compared using chi-square tests. Significant p-values are depicted in bold. BMI, Body mass index; WHR, waist hip ratio; HbA1c, glycated hemoglobin; eGFR MDRD, Glomerular filtration ratio calculated according to MDRD; ASAT, Aspartate aminotransferase; ALAT, Alanine aminotransferase; GGT, Gamma-glutamyltransferase; FFA, Free fatty acids; pro-ANP, pro-atrial natriuretic peptide; PPI, Proton-pump-inhibitors; PPI, Protein-pump-inhibitors; aHEI, alternative Healthy Eating Index; DASH: Dietary Approach to Stop Hypertension.

Supplementary Data 2:

Gut microbiome compositional and functional variance explained by host variables, univariate analyses. R2 (explained variance) and P-values derived from univariate ADONIS analysis of the Bray-Curtis dissimilarity indices of genus, MGS, GMM and KEGG pathways, respectively, using key environmental variables as predictors (e.g., diet, medication, demographics, lifestyle, preconditions, clinical and stool characteristics) and 20,000 permutations in the MetaCardis healthy individuals (n = 200). MGS: metagenomic species, GMM: gut metabolic modules, ADONIS: Analysis of variance using distance matrices. Multiple testing correction was done using Benjamini-Hochberg method and FDR ≤ 0.1 was considered significant.

Supplementary Data 3:

Host variables affect the gut microbiome, metabolome and phenome in healthy individuals. Associations among host variables (e.g., diet, medication, demographics, lifestyle, preconditions and stool characteristics) and gut microbiome composition (i.e., genus, MGS) and functions (i.e., GMM, KEGG pathways) and metabolome tested using Spearman correlation in the MetaCardis healthy individuals (n = 200-275). Multiple testing correction was done using Benjamini-Hochberg method and FDR ≤ 0.1 was considered significant. MGS: metagenomic species, GMM: gut metabolic modules; KEGGp: KEGG pathways; BH: Benjamini-Hochberg; FDR: false-discovery rate.

Supplementary Table 4:

Microbiome affects the phenome in healthy individuals. Multivariate (CA-PLS) models explaining (R2) and predicting (q2 as the goodness of prediction) the proportion of variance of clinical variables using gut microbiome composition (i.e., genus, MGS) and functions (i.e., GMM, KEGGp) as predictors and demographics (i.e., age, sex and country) as covariates in the MetaCardis healthy individuals (n = 275). Clinical data were imputed using kNN=10 for this analysis. Multiple testing correction was done using Benjamini-Hochberg method and FDR ≤ 0.1 considered significant. CA-PLS: Covariate-adjusted partial

least square regression, MGS: metagenomic species, GMM: gut metabolic modules, KEGGp: KEGG pathways. BH: Benjamini-Hochberg; FDR: false-discovery rate.

Supplementary Data 5:

Gut microbial functions associate with circulating proANP levels in healthy individuals. Associations among clinical variables and gut microbiome composition (i.e., MGS) and functions (i.e., GMM, KEGG pathways) tested using demographics adjusted linear models in the MetaCardis healthy individuals (n = 255). Partial spearman correlations were used for MGS whereas linear regression using rank normalized data was used for GMM and KEGG pathways. Multiple testing correction was done using BH criteria for each metagenomic data category and FDR ≤ 0.1 was considered significant. MGS: metagenomic species; GMM: gut metabolic modules; BH: Benjamini-Hochberg; FDR: false-discovery rate.

Supplementary Data 6:

Gut microbial functions associated with circulating proANP levels are driven by fecal microbial load in healthy individuals. Associations among circulating proANP levels and gut microbiome GMM and KEGG pathways tested using two models i.e., model 1: demographics adjusted and model 2: demographics and fecal microbial load adjusted, in the MetaCardis healthy individuals (n = 255). Linear regression using rank normalized data followed by multiple testing correction using BH criteria per metagenomic data category, and FDR ≤ 0.1 was considered significant. GMM: gut metabolic modules; BH: Benjamini-Hochberg; FDR: false-discovery rate.

Supplementary Data 7:

Mediation analyses testing gut microbial functions (GMMs) as mediators of the fecal microbial load-proANP associations in healthy individuals. ACME, ADE and total effects, related P-values were derived from demographics-adjusted mediation analyses (using mediation package in R) for each of 78 GMMs that associated with proANP in demographics-adjusted linear models (n =255). Multiple testing correction was done using Benjamini-Hochberg method and FDR ≤ 0.1 was considered significant. GMM: gut metabolic modules; ACME: average causal mediation effects; ADE: average direct effects.

Supplementary Data 8:

Mediation analyses testing gut microbial functions (KEGG pathways) as mediators of the fecal microbial load-proANP associations in healthy individuals. ACME, ADE and total effects, related P-values were derived from demographics-adjusted mediation analyses (using mediation package in R) for each of 168 KEGG pathways that associated with proANP in demographics-adjusted linear models (n =255). Multiple testing correction was done using Benjamini-Hochberg method and FDR ≤ 0.1 was considered significant. GMM: gut metabolic modules; ACME: average causal mediation effects; ADE: average direct effects.

Supplementary Data 9:

Metabolites associated with plasma proANP concentrations in healthy individuals. Associations among circulating proANP levels and metabolome tested using linear models adjusted for demographics (i.e., age, sex and country) in the MetaCardis healthy individuals (n = 230-274). Effect sizes (standardised beta)

and significance level are reported (linear regression using ranknormalized data; multiple testing corrections done using Benjamini-Hochberg method and FDR ≤ 0.1 considered significant.

Supplementary Data 10:

Microbiome affects the metabolome in healthy individuals. Multivariate (CA-PLS) models explaining (R2) and predicting (q2 as the goodness of prediction) the proportion of variance of metabolome using gut microbiome composition (i.e., genus, MGS) and functions (i.e., GMM, KEGGp) as predictors and demographics (i.e., age, sex and country) as covariates in the MetaCardis healthy individuals (n = 247-275). Multiple testing correction was done using Benjamini-Hochberg method and FDR ≤ 0.1 considered significant. CA-PLS: Covariate-adjusted partial least square regression, MGS: metagenomic species, GMM: gut metabolic modules, KEGGp: KEGG pathways. BH: Benjamini-Hochberg; FDR: false-discovery rate. Only metabolites exhibiting sparsity<0.2 were retained in the analysis.

Supplementary Data 11:

Microbiome associates with key metabolites and kidney-heart variables in healthy individuals. Associations among metagenomic species and key metabolites derived from phenylalanine-tyrosine metabolism as well as key clinical variables tested using demographics-adjusted Spearman correlations in the MetaCardis healthy individuals. Effect sizes (Spearman rho) and significance level of the tested variables are reported (n = 247-274; multiple testing corrections done using Benjamini-Hochberg method and FDR ≤ 0.1 considered significant. CRE: 4-cresol; PCS:4-cresyl sulfate; PCG:4-cresyl glucuronide; PAA: phenylacetate; PAG: phenylacetylglutamine; PAC: phenylacetylcarnitine; HC: 3-phenylpropionate; CG: cinnamoylglycine; PS: phenol sulfate; HPLA: 3-(4-hydroxyphenyl)-lactate; VLA: vanillactate; proANP: pro-atrial natriuretic peptide; eGFR: glomerular filtration ratio calculated using MDRD formula; SBP: systolic blood pressure; VFR: visceral fat rating; TFM: total fat mass.

Supplementary Data 12:

Confounders analyses in the MetaCardis population. List of host variables included in the confounder analyses for the metabolically healthy controls and individuals with CMD in the MetaCardis population, respectively.

Supplementary Data 13:

Confounder-controlled associations among gut microbiome, key metabolites and kidney-heart variables identified in the healthy individuals and replicated in those with CMD within the MetaCardis population. List of associations among gut microbiome features (i.e., ecological, taxonomy and functions), key metabolites derived from phenylalanine-tyrosine metabolism and kidney-heart variables (i.e., proANP, eGFR, creatinine) that exhibited FDR ≤ 0.1 and a deconfounded status in metadeconfoundR analysis in the MetaCardis healthy individuals (n = 247-275) followed by their replication in individuals with CMD (n = 1562-1585). Features listed in Supplementary Data 12 were included for confounder testing of each of these groups, respectively. Effects sizes were derived from demographics-adjusted linear models (i.e., Spearman's correlation for ecological, genus and MGS; linear regression using ranknormalized data for GMM, KEGG pathways and metabolites). Multiple testing corrections were done using Benjamini-

Hochberg method and FDR ≤ 0.1 was considered significant. Replication was ascertained by 1) effect size alignment between two groups, ii) FDR ≤ 0.1 and iii) deconfounded status. CRE: 4-cresol; PCS:4-cresyl sulfate; PCG:4-cresyl glucuronide; PAA: phenylacetate; PAG: phenylacetylglutamine; PAC: phenylacetylcarnitine; HC: 3-phenylpropionate; CG: cinnamoylglycine; PS: phenol sulfate; HPLA: 3-(4-hydroxyphenyl)-lactate; VLA: vanillactate; proANP: pro-atrial natriuretic peptide; eGFR: glomerular filtration ratio calculated using MDRD formula, creat: creatinine; MGS: metagenomic species, GMM: gut metabolic modules.

Supplementary Data 14:

Mediation analyses testing metabolites as mediators of the microbiome-eGFR associations in healthy individuals. ACME, ADE, total effects and related P-values were derived from demographics-adjusted mediation analyses (using mediation package in R) for each deconfounded microbiome-metabolite association listed in Supplementary Data 13, where microbiome feature was treated as the independent variable, metabolite as the mediator and eGFR as the outcome variable in the MetaCardis healthy individuals (n =246-271). Multiple testing correction (per microbiome feature group) was done using Benjamini-Hochberg method and FDR ≤ 0.1 was considered significant. ACME: average causal mediation effects; ADE: average direct effects.

Supplementary Data 15:

Mediation analyses testing metabolites as mediators of the microbiome-proANP associations in healthy individuals. ACME, ADE, total effects and related p-values were derived from demographics-adjusted mediation analyses (using mediation package in R) for each deconfounded microbiome-metabolite association listed in Supplementary Data 13, where microbiome feature was treated as the independent variable, metabolite as the mediator and proANP as the outcome variable in the MetaCardis healthy individuals (n =230-253). Multiple testing correction (per microbiome feature group) was done using Benjamini-Hochberg method and FDR ≤ 0.1 was considered significant. ACME: average causal mediation effects; ADE: average direct effects.

Supplementary Data 16:

Genetic instruments used in the Mendelian Randomization analyses.

Supplementary Data 17:

Univariate Mendelian Randomization analyses of circulating metabolites and kidney function (eGFR). Genetic associations for key metabolites were derived from the CLSA except for 4-cresol, which were derived from the EGEA (see Methods) and eGFR from the CKDGen. For related sctter and funnel plots please see Supplementary Figures 15 and 17. Table A represents the estimates from IVW pre- and post-outlier correction, B, sensitivity analyses pre- and C, post -outlier correction, respectively. FDR (Table A) was calculated for all metabolites pre- and post-outlier correction separately. CRE: 4-cresol; PCS:4-cresyl sulfate; PCG:4-cresyl glucuronide; PAA: phenylacetate; PAG: phenylacetylglutamine; PAC: phenylacetylcarnitine; HC: 3-phenylpropionate; CG: cinnamoylglycine; PS: phenol sulfate; HPLA: 3-(4-hydroxyphenyl)-lactate; VLA: vanillactate.

Supplementary Data 18:

Univariate Mendelian Randomization analyses of eGFR and circulating metabolites. Genetic associations for key metabolites were derived from the CLSA except for 4-cresol, which were derived from the EGEA (see Methods) and for eGFR from the CKDGen. For related sctter and funnel plots please see Supplementary Figures 16 and 17. Table A represents the estimates from IVW pre- and post-outlier correction, B, sensitivity analyses pre- and C, post -outlier correction, respectively. FDR (Table A) was calculated for all metabolites pre- and post-outlier correction separately. CRE: 4-cresol; PCS:4-cresyl sulfate; PCG:4-cresyl glucuronide; PAA: phenylacetate; PAG: phenylacetylglutamine; PAC: phenylacetylcarnitine; HC: 3-phenylpropionate; CG: cinnamoylglycine; PS: phenol sulfate; HPLA: 3-(4-hydroxyphenyl)-lactate; VLA: vanillactate.

Supplementary Data 19:

Univariate Mendelian Randomization analyses of circulating metabolites and NPPA. Genetic associations for metabolites were derived from the CLSA except for 4-cresol, which were derived from the EGEA (see Methods) and for NPPA from the deCODE study. For related sctter and funnel plots please see Supplementary Figures 18 and 20. Table A represents the estimates from IVW pre- and post-outlier correction, B, sensitivity analyses pre- and C, post -outlier correction, respectively. FDR (Table A) was calculated for all metabolites pre- and post-outlier correction separately. CRE: 4-cresol; PCS:4-cresyl sulfate; PCG:4-cresyl glucuronide; PAA: phenylacetate; PAG: phenylacetylglutamine; PAC: phenylacetylcarnitine; HC: 3-phenylpropionate; CG: cinnamoylglycine; PS: phenol sulfate; HPLA: 3-(4-hydroxyphenyl)-lactate; VLA: vanillactate.

Supplementary Data 20:

Univariate Mendelian Randomization analyses of NPPA on circulating metabolites. Genetic associations for metabolites were derived from the CLSA except for 4-cresol, which were derived from the EGEA (see Methods) and for NPPA from the deCODE study. For related sctter and funnel plots please see Supplementary Figures 19 and 20. Table A represents the estimates from IVW pre- and post-outlier correction, B, sensitivity analyses pre- and C, post -outlier correction, respectively. FDR (Table A) was calculated for all metabolites pre- and post-outlier correction separately. CRE: 4-cresol; PCS:4-cresyl sulfate; PCG:4-cresyl glucuronide; PAA: phenylacetate; PAG: phenylacetylglutamine; PAC: phenylacetylcarnitine; HC: 3-phenylpropionate; CG: cinnamoylglycine; PS: phenol sulfate; HPLA: 3-(4-hydroxyphenyl)-lactate; VLA: vanillactate.

Supplementary Data 21:

Univariate bi-directional Mendelian Randomization analyses of eGFR-NPPA axis. Genetic associations for eGFR were derived from the CKDGen and for NPPA from the deCODE study. For related scatter and funnel plots please see Supplementary Figure 21.
